# A Robust Tuberculosis Diagnosis Using Chest X-Rays Based on a Hybrid Vision Transformer and Principal Component Analysis

**DOI:** 10.3390/diagnostics14232736

**Published:** 2024-12-05

**Authors:** Sameh Abd El-Ghany, Mohammed Elmogy, Mahmood A. Mahmood, A. A. Abd El-Aziz

**Affiliations:** 1 Department of Information Systems, College of Computer and Information Sciences, Jouf University, Aljouf, P.O. Box 2014, Sakaka 72388, Saudi Arabia; mamahmood@ju.edu.sa (M.A.M.); aaeldamarany@ju.edu.sa (A.A.A.E.-A.); 2 Information Technology Department, Faculty of Computers and Information, Mansoura University, Mansoura P.O. Box 35516, Egypt; melmogy@mans.edu.eg

**Keywords:** tuberculosis, vision transformer (ViT), deep learning, machine learning, CAD, predict, chest X-rays

## Abstract

**Background**: Tuberculosis (TB) is a bacterial disease that mainly affects the lungs, but it can also impact other parts of the body, such as the brain, bones, and kidneys. The disease is caused by a bacterium called Mycobacterium tuberculosis and spreads through the air when an infected person coughs or sneezes. TB can be inactive or active; in its active state, noticeable symptoms appear, and it can be transmitted to others. There are ongoing challenges in fighting TB, including resistance to medications, co-infections, and limited resources in areas heavily affected by the disease. These issues make it challenging to eradicate TB. Objective: Timely and precise diagnosis is essential for effective control, especially since TB often goes undetected and untreated, particularly in remote and under-resourced locations. Chest X-ray (CXR) images are commonly used to diagnose TB. However, difficulties can arise due to unusual findings on X-rays and a shortage of radiologists in high-infection areas. **Method**: To address these challenges, a computer-aided diagnosis (CAD) system that uses the vision transformer (ViT) technique has been developed to accurately identify TB in CXR images. This innovative hybrid CAD approach combines ViT with Principal Component Analysis (PCA) and machine learning (ML) techniques for TB classification, introducing a new method in this field. In the hybrid CAD system, ViT is used for deep feature extraction as a base model, PCA is used to reduce feature dimensions, and various ML methods are used to classify TB. This system allows for quickly identifying TB, enabling timely medical action and improving patient outcomes. Additionally, it streamlines the diagnostic process, reducing time and costs for patients and lessening the workload on healthcare professionals. The TB chest X-ray dataset was utilized to train and evaluate the proposed CAD system, which underwent pre-processing techniques like resizing, scaling, and noise removal to improve diagnostic accuracy. **Results**: The performance of our CAD model was assessed against existing models, yielding excellent results. The model achieved remarkable metrics: an average precision of 99.90%, recall of 99.52%, F1-score of 99.71%, accuracy of 99.84%, false negative rate (FNR) of 0.48%, specificity of 99.52%, and negative predictive value (NPV) of 99.90%. **Conclusions**: This evaluation highlights the superior performance of our model compared to the latest available classifiers.

## 1. Introduction

All cells in our bodies need oxygen to survive. This essential element is carried to the cells through a coordinated effort between the heart and lungs, known as the pulmonary circuit. This process ensures that the blood carries the body’s oxygen needs. When the heart detects blood with low oxygen levels, it transports it to the lungs for purification and oxygenation. The lungs also play a vital role in adjusting the incoming oxygen to match the body’s temperature and humidity levels. Additionally, they remove carbon dioxide during exhalation to maintain a healthy balance of gases in the body [1]. Lung disorders frequently result in considerable discomfort and distress as they interfere with individuals’ breathing patterns, causing breathlessness and associated symptoms. These disorders are generally classified into three main categories: bronchitis, circulatory conditions, and lung tissue diseases that affect the lungs. Bronchitis and lung tissue diseases hinder lung expansion [2]. In cases where issues with blood vessels in the lungs lead to inflammation or the formation of blood clots, the regular breathing process becomes challenging [3].

TB is a severe lung disease that significantly damages humanity. The bacterium Mycobacterium tuberculosis is responsible for causing TB. In dealing with TB, developing a thorough treatment plan for patients is necessary. The lungs are typically the main part of the body that gets affected by this illness, but it can also influence different areas, such as the brain, bones, stomach, and kidneys [4]. TB is an infectious disease where individuals spread disease-causing bacteria into the air when they cough or sneeze. Even a small number of these bacteria can sufficiently infect a healthy person. TB poses a significant health challenge globally, especially in developing nations where it is widespread, such as India, China, South Africa, Indonesia, Pakistan, Bangladesh, the Philippines, and Nigeria. TB is predominantly found in the Western Pacific, Africa, and Southeast Asia, making up the majority (43%, 25%, and 18%, respectively) of cases globally. Smaller proportions are seen in Europe (2.3%), the Americas (3.0%), and the Eastern Mediterranean (8.3%) [5].

In 2019, TB overtook Human Immunodeficiency Virus (HIV) and Acquired Immune Deficiency Syndrome (AIDS) as the most lethal infectious disease, responsible for the highest number of fatalities from a single source. According to World Health Organization (WHO) data, approximately 10 million people (5.6 million men, 3.2 million women, and 1.2 million children) worldwide were affected by TB in 2019 [5]. The death toll due to TB has been on the rise primarily due to limited access to medical treatment. A recent spike in TB-related deaths has been observed, with global fatalities reaching 1.5 million in 2020, exacerbated by the impact of COVID-19. This marks the first increase in year-on-year TB deaths since 2005 [6].

Common symptoms of active TB include persistent cough, fever, night sweats, weight loss, shivering, feeling out of breath, tiredness, and a reduced desire to eat [7]. Detecting TB promptly is essential for patients to receive adequate treatment, enhance survival rates, and stop the continued transmission of Mycobacterium TB bacteria. Identifying unique traits and indicators in TB patients during the initial phase can be challenging [8]. Identifying TB early necessitates comprehensive healthcare coordination, proficient medical professionals, and experienced radiologists. Individuals can undergo clinical treatment when an accurate diagnosis is established and suitable medical care is provided initially. Consequently, advancing integrated healthcare services is the key to successfully treating TB patients [9].

TB can be diagnosed through various tests, including skin, blood, and imaging studies. Treatment typically involves a combination of antibiotics taken over several months to kill the bacteria and prevent the development of drug-resistant strains. The most reliable method for detecting TB cases is isolating microorganisms causing the infection. While this approach can offer a high level of precision, it tends to have a relatively lower level of accuracy. The process of obtaining test results can be extended, sometimes up to three weeks. Alternative approaches in scientific methods, such as immunological assays and molecular biology techniques, have advantages and disadvantages. For example, immunological tests are praised for their simplicity and quickness in carrying out experiments. However, they have limited sensitivity and specificity, reducing usage in situations involving chronic TB infections [10].

Polymerase chain reaction (PCR) is widely used to amplify nucleic acids. It is a very effective test for detecting TB in clinical samples, such as sputum, blood, bone marrow, and biopsy specimens. However, PCR methods can be expensive and unavailable in all healthcare facilities. It is essential to carefully consider the pros and cons of other diagnostic techniques when deciding on the best approach for diagnosing TB. Challenges, such as the time needed for processing, additional costs, the requirement for expert molecular professionals, and establishing laboratory facilities, all act as obstacles that impede the execution of rapid molecular diagnostic tests [11].

In recent years, chest X-ray (CXR) images have significantly increased in identifying and screening for pulmonary TB. These images provide detailed information about the body’s structure, revealing specific visual indicators associated with TB, such as nodules, cavities, and infiltrates. However, the interpretation of CXR images by radiologists may vary due to individual judgment, differing expertise, and experience, leading to potential discrepancies. The variations in diagnosing illnesses from X-rays highlight the diversity in the analysis [12,13]. CXR scans showing TB symptoms are often misinterpreted as other conditions with similar visual characteristics. This misdiagnosis can lead to incorrect treatment, worsening the patient’s health. Additionally, regions with limited resources, mainly rural areas in various countries, face a shortage of skilled radiologists, further complicating the situation [14,15]. Therefore, combining advanced technology with medical knowledge and protocols is crucial to address this issue effectively.

Artificial intelligence (AI) has made remarkable progress in recent decades and has extended into different industries. In the healthcare sector, its use has led to rapid progress in many research areas, enabling researchers to explore new frontiers [4]. The use of computer-aided diagnosis (CAD) technology to analyze medical images is widespread. Numerous CAD systems have been developed specifically to examine CXR images for signs of TB. Advanced deep learning (DL) methods are becoming essential in medical image-based diagnostic systems. These approaches use convolutional neural networks (CNN), which consist of complex feature extractors that support various applications. While developing the CAD system for detecting TB, it is crucial to train the system using a wide variety of data, including images with noise. The primary goal of the diagnostic system is to examine CXR images with greater accuracy than radiologists. Developing an AI tool to precisely predict TB results from CXR images is essential in densely populated nations to combat the limited number of radiologists [16].

This research presents a CAD system that leverages the use of the ViT to precisely detect TB in CXR images. This innovative hybrid CAD approach combines ViT with PCA and ML techniques for classifying TB. In the hybrid CAD system, ViT is utilized for extracting deep features, PCA is employed to reduce the dimensions of these features, and various ML methods are applied to classify TB. The innovative CAD system enables the rapid and precise identification of TB, promoting timely interventions and enhancing patient outcomes. Furthermore, it simplifies the diagnostic process, minimizing both time and expenses for patients while easing the workload for healthcare professionals. The CAD system can be integrated as a decision-support tool within existing radiology workflows. It serves as a secondary opinion system, flagging cases for further examination by radiologists rather than making standalone diagnoses. The effectiveness of the proposed CAD model was assessed using the TB Chest X-ray dataset [17,18]. The TB Chest X-ray dataset underwent pre-processing using resizing, scaling, and noise removal techniques to improve diagnostic accuracy. The dataset was divided into 80% for training and 20% for testing. Our CAD model was compared to other existing models, showcasing outstanding results. The model exhibited impressive metrics, including an average precision of 99.90%, recall of 99.52%, F1-score of 99.71%, accuracy of 99.84%, FNR of 0.48%, specificity of 99.52%, and NPV of 99.90%. This assessment highlights the exceptional accuracy achieved by fine-tuning our model compared to the most recent classifiers. The primary contributions of this research are as follows:

▪ We proposed a hybrid CAD system that combines a ViT to extract deep features from CXR images, uses PCA to reduce the dimensions of these features, and applies various ML methods to classify TB in its early stages. ViT gathers the contextual data and long-range dependencies necessary for precise disease classification.
▪ This CAD system enables swift detection of TB, facilitating timely medical intervention and enhancing patient outcomes. Moreover, it simplifies the diagnostic procedure, leading to time and cost efficiencies for patients while reducing the burden on healthcare providers.
▪ We compared the proposed ViT model with several leading pre-trained DL models, including DenseNet121, MobileNet, ResNet101V2, Xception, and InceptionV3. Additionally, we evaluated them alongside five ML models, which include Random Forest (RF), Extreme Gradient Boosting (XGB), Decision Tree (DT), Support Vector Machine (SVM), and Adaptive Boosting (AdaBoost).
▪ The proposed system achieved an average precision of 99.90%, recall of 99.52%, F1-score of 99.71%, accuracy of 99.84%, FNR of 0.48%, specificity of 99.52%, and NPV of 99.90%.

The rest of the paper structure is organized into four sections. Section 2 provides a review of TB diagnostic models found in the literature. Section 3 details the proposed methodology. Section 4 showcases the experimental results. Section 5 outlines the conclusion of the proposed model.

## 2. Literature Review

Medical image analysis is considered a hot research topic that can detect and diagnose many disorders using different imaging modalities. This can assist doctors in reaching a fast and accurate diagnosis for different disorders. TB diagnosis using CXR analysis grabs the attention of many researchers. For example, Acharya et al. [4] introduced a method known as progressive resizing to train models that can automatically infer TB from CXR images. They utilized training on ImageNet fine-tuned Normalization-Free Networks (NFNets) with the Score-CAM algorithm to pinpoint specific regions in CXR images for detailed diagnosis. This approach aimed at providing accurate diagnostic results for binary and multiclass classification tasks. The models developed through this methodology achieved accuracy equal to 96.91%, sensitivity equal to 91.81%, specificity equal to 98.42%, and the area under the curve (AUC) equal to 99.38% when tested on a diverse multiclass dataset comprising TB Chest X-ray, Montgomery, and Shenzhen images. Furthermore, these models have achieved 98% AUC and 96% accuracy in binary classification scenarios.

Huy and Lin [10] created the CBAMWDnet model to detect TB in CXR images. This model integrates the convolutional block attention module (CBAM) with the Wide Dense Net (WDnet) design to effectively capture spatial and contextual details within the images. After thoroughly evaluating a large dataset of CXR images, CBAMWDnet demonstrated superior performance compared to existing models. The model was assessed on a comprehensive dataset, including TB Chest X-ray, Montgomery, and Shenzhen. It achieved results with an accuracy of 98.80%, sensitivity of 94.28%, precision of 98.50%, specificity of 95.70%, and F1-score of 96.35%. Additionally, CBAMWDnet exhibited strong generalization capabilities by consistently delivering reliable results across multiple datasets.

Le et al. [19] utilized TB Chest X-ray, Montgomery, and Shenzhen datasets that were previously made public. These datasets were adjusted to ensure consistent input data for training purposes. In the deep neural networks (DNNs) field, they initially chose VGG16 as the base network architecture. Later, they also investigated EfficientNet-B7, MobileNetV3, DenseNet121, and RegNetY040. Their analysis of different Chest X-ray datasets revealed that specific models produced superior results. DenseNet121 performed exceptionally well with the Shenzhen dataset, while EfficientNet-B7 showed remarkable performance with the India dataset. MobileNetV3 stood out in the TB Chest X-ray dataset (98.35%) and Montgomery dataset, achieving outstanding accuracy and F1-score metrics.

Visuña et al. [20] improved the performance of CNNs by implementing TL and data augmentation techniques. They also created and evaluated two different CNN-ensembles: Stacking and Voting. Their system was designed to be seamlessly integrated into a CAD system, allowing for automated diagnostics as a supporting tool or an initial assessment before consulting healthcare professionals. The study results showed remarkable improvements, with the stacking ensemble achieving an accuracy rate of 99% and the voting ensemble achieving a commendable accuracy rate of 98%.

Goswami et al. [21] introduced a method to detect TB by analyzing CXR images from the TB Chest X-ray dataset using CNNs. To develop the system, the study examined 1196 CXR images, including TB-negative and TB-positive samples. The system was trained to identify specific features in TB CXR images, enabling early TB detection for timely treatment. It achieved an impressive average precision rate of 93.4%, with high recall and precision rates of 94.1%, highlighting its effectiveness in distinguishing between TB-negative and TB-positive cases. Moreover, the model showed a specificity of 91.49%, a sensitivity of 96.85%, and an F1-score of 94.1%. Overall, the model exhibited an accuracy of 94%.

Sharma et al. [22] conducted a study where they trained a UNet segmentation model using 704 CXR images from Montgomery County and Shenzhen Hospital datasets. They then applied this model to segment lung areas in 1400 TB and control CXR scans from the NIAID TB portal program dataset. Subsequently, they used the Xception model to classify the segmented lung regions into TB and normal categories. Furthermore, they analyzed the model’s visualization capabilities using Grad-CAM to identify TB anomalies in CXR images from a radiological perspective. The UNet model achieved notable performance metrics, including an accuracy of 96.35%, a Jaccard index of 90.38%, a Dice similarity coefficient (DSC) of 94.88%, and an AUC value of 99% for segmentation. In contrast, the Xception model demonstrated outstanding results with a classification accuracy of 99.29%, precision of 99.30%, recall of 99.29%, F1 score of 99.29%, and an AUC value of 99.9%. Interestingly, the Grad-CAM heatmaps highlighted TB cases showing consistent patterns with lesions mainly concentrated in the upper lung regions.

Maheswari et al. [23] proposed a shallow CNN to screen TB from CXR images of the TB Chest X-ray dataset. The primary goal was to offer precise interpretations for accurate diagnoses. This model was structured with four convolution-max-pooling layers, utilizing optimized hyperparameters to enhance performance through Bayesian optimization techniques. The model delivered exceptional outcomes, with a maximum F1-score, specificity sensitivity, and accuracy, reaching 95%. Moreover, the proposed model’s receiver operating characteristic (ROC) curve showcased an impressive peak AUC value of 97.6%. To ensure transparency and interpretability, the authors employed class activation maps (CAM) and local interpretable model-agnostic explanations (LIME) systems to evaluate the model, contrasting it with a cutting-edge pre-trained neural network such as DenseNet.

In contrast to the studies mentioned earlier that employed CNNs, our method leveraged ViT. ViTs and CNNs represent two separate categories of structures applied in image-related assignments. Despite CNNs being the preferred choice for an extended period, ViTs have garnered attention lately because of their various benefits, such as using the self-attention mechanism to analyze connections across all elements of an image at once. This broad context comprehension is beneficial for recognizing far-reaching connections and intricate designs. However, CNNs mainly depend on local receptive fields. This implies that they analyze small portions of the image sequentially. While deeper layers can grasp a broader context, their early layers remain fundamentally focused on local information. The scalability of ViTs is evident when more data and larger models are introduced. CNNs, although capable of scaling, typically need more complex structural adjustments (such as utilizing various layers and operations) to make notable enhancements.

## 3. Materials and Methods

This research presents a hybrid CAD system crafted to accurately identify TB in CXR images using the ViT model, PCA, and various ML techniques. The approach includes training and assessing the CAD system on the TB Chest X-ray dataset, allowing it to accurately categorize images as either TB-positive or normal with remarkable precision. When given a CXR image as input, the model harnesses its sophisticated DL capabilities to predict the image.

### 3.1. Dataset

A collaborative effort between researchers at Qatar University in Doha, Qatar, and the University of Dhaka in Bangladesh, alongside partners in Malaysia and medical professionals from Hamad Medical Corporation and Bangladesh, has led to the development of the TB Chest X-ray dataset. This dataset contains images of individuals with TB and those with normal cases. The current database release includes 700 TB images and 3500 images of normal cases [17,18]. The TB Chest X-ray dataset was generated using information from three databases that are open to the public, as shown in Table 1:

1. NLM dataset [24]: The National Library of Medicine (NLM) in the United States has made two CXR datasets available for public use: the Shenzhen Hospital (SH) dataset and the Montgomery County (MC) dataset. The SH dataset includes 336 CXR images of patients with TB and 324 images of individuals without TB. The MC dataset contains 138 CXR images, of which 58 indicate TB infection, while the remaining 80 images belong to individuals with no visible health concerns. Although the datasets are intended for a global audience, they are primarily focused on specific geographic regions where the prevalence of tuberculosis is notably high.
2. Belarus dataset [25]: The data were obtained from a medical institution in Belarus and included CXR scans of individuals screened for lung-related conditions, such as TB. The dataset contains 306 images representing 169 unique individuals diagnosed with TB. While it offers a more specific geographic focus, it remains an essential representation of Eastern European demographics.
3. RSNA dataset [26]: The Radiological Society of North America (RSNA) dataset for the pneumonia detection challenge includes around 30,000 CXR images. This dataset is diverse, covering a range of conditions, but it mainly reflects contributions from North America and worldwide. Of these images, 10,000 showed normal findings, while the remaining images displayed various abnormalities, such as lung opacity. Additionally, 3094 regular images were selected from this dataset.

The NLM and RSNA datasets comprise a combination of healthy and pathological cases, whereas the Belarus dataset predominantly features pathological cases due to its clinical setting. RSNA’s dataset is among the largest available and includes images sourced from various facilities, which improves the diversity of equipment and imaging conditions. Data collection for NLM and RSNA adhered to strict ethical guidelines, ensuring informed consent when necessary and the anonymization of patient data. Similarly, the Belarus dataset followed institutional protocols for research data collection and was also anonymized to safeguard patient privacy [24,25,26].

**Figure 1:**
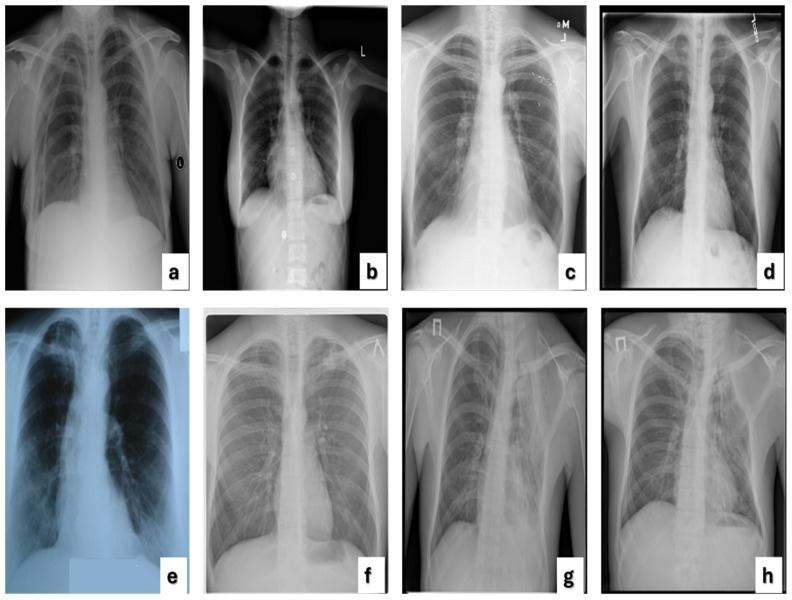
Some examples of the TB and normal CXR images: (**a**–**d**) normal cases and (**e**–**h**) TB cases.

In this research, 80% of the complete TB Chest X-ray dataset was used for training, while the remaining 20% was specifically set aside for testing, as illustrated in Table 2. Figure 1 displays images of TB CXR and normal CXR. The three datasets, NLM, Belarus, and RSNA, were merged and divided into training and test sets through a stratified sampling method, maintaining a consistent class distribution (normal and TB cases) across all subsets. Additionally, we ensured that images from the same patient were included in only one subset to prevent data leakage.

### 3.2. Proposed Methodology

**Figure 2:**
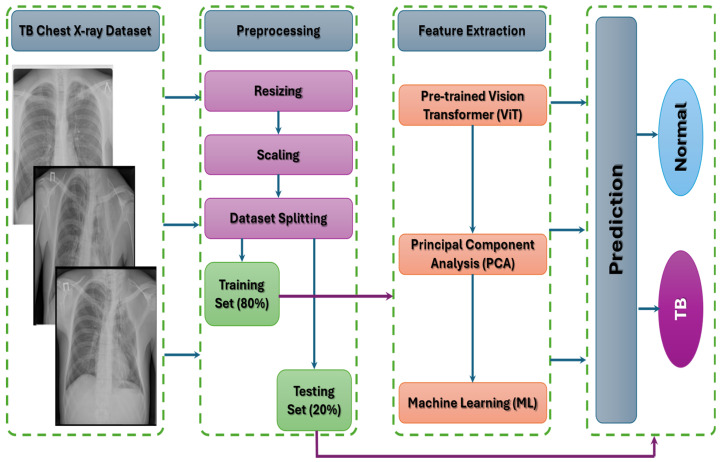
The proposed model architecture for TB diagnosis based on a hybrid vision transformer and principal component analysis with machine learning.

We introduced a hybrid fine-tuned CAD model employing the ViT with PCA and ML techniques (RF, XGB, DT, SVM, and AdaBoost) to classify TB. In the hybrid CAD system, ViT was utilized for extracting deep features, PCA was employed to reduce the dimensions of these features, and the ML methods (RF, XGB, DT, SVM, and AdaBoost) were applied to classify TB. Our meticulously crafted CAD model underwent thorough testing using the TB Chest X-ray dataset. The assessment demonstrated the exceptional performance of our model, outperforming recent alternatives due to meticulous fine-tuning. Figure 2 illustrates the overall architecture of our CAD model, and Algorithm 1 provides an overview of the fine-tuning algorithm for the proposed ViT model and five DL techniques. The proposed CAD model encompasses the following stages:

**Phase 1: The TB Chest X-ray dataset pre-processing**: CXR images underwent pre-processing to boost the performance of the proposed CAD system. This involved resizing, scaling, and noise removal of the CXR images.

**Phase 2: The TB Chest X-ray dataset splitting**: After pre-processing the CXR images, we divided the TB Chest X-ray dataset into 80% for training (3360 CXR images) and 20% for testing (840 CXR images).

**Phase 3: Pre-training for the six DL models the ViT**: The six DL algorithms were pre-trained on the ImageNet dataset to extract the deep features from CXR images.

**Phase 4: Fine-tuning the five ML models**: The five ML models were adjusted using the training set of the TB Chest X-ray dataset to accurately classify TB.

**Phase 5**: Evaluation of the five ML models: Various metrics, including precision, recall, FNR, NPV, and accuracy, were used to evaluate how well the proposed CAD models performed on the test dataset.
**Algorithm 1: The Proposed CAD Algorithm**1**Input** → TB Chest X-ray Dataset *TBD*.2**Output** ← Fine-tuned CAD Model using ViT TB diagnosis.3**BEGIN**4   **STEP 1: Pre-processing of Images**
5 **FOR EACH** CXR image **IN** the *TBD* **DO**6           *Remove* noise.7           *Resize* 224 × 224 pixels.8           *Scaling* pixel to range [0, 1].9       **END FOR**
10   **STEP 2: Splitting *TBD***
11     **SPLIT** *TBD* **INTO**12      *Training set* → 80% *of TBD*.13      *Test set* → 20% *of TBD*.14   **STEP 3: Six Models Pre-training**
15      **FOR EACH DL IN [ViT, DenseNet121, MobileNet, ResNet101V2, Xception] DO**
16           *Load DL*.17           *Pre-train DL* on the ImageNet dataset.18           *Extract* deep features from CXR images.19      **END FOR**
20   **STEP 4: Five ML Models Fine-Tunning**
21      **FOR EACH ML IN [RF, XGB, DT, SVM, and AdaBoost] DO**
22           *Fine-tune* ML on the training set. 23      **END FOR**
24   **STEP 5: Model Evaluation**
25      **FOR EACH ML IN [RF, XGB, DT, SVM, and AdaBoost] DO**
26       *Evaluate* the effectiveness of ML on the test set of the fine-tuned CAD model.27      **END FOR**
28       Keep the accuracy of the fine-tuned CAD model.29**END**

#### 3.2.1. Image Pre-Processing

Image pre-processing involves the methods utilized to refine CXR images before inputting them into computer vision algorithms. This pre-processing is essential for enhancing the quality of CXR images and optimizing the performance of CAD models. The typical pre-processing steps for CXR images include resizing, normalization, and noise reduction [27].

##### Resizing

Resizing images is a fundamental step in image preprocessing, particularly for tasks involving neural networks, which often require fixed-sized input images. This process involves scaling the image’s dimensions while preserving critical visual features. For an image with original dimensions w × h (width by height), resizing to new dimensions w′×h′ is typically achieved using interpolation methods such as nearest-neighbor, bilinear, or bicubic interpolation. The aspect ratio, defined as w’/h’, can be preserved by adjusting only one dimension while maintaining the ratio for the other, or it can be altered, which may introduce distortion. If maintaining the aspect ratio is critical, padding may be applied to achieve the desired output size, typically using techniques like zero padding or border reflection. The trade-off in resizing involves balancing reduced computational cost and the potential loss of fine details in the image [27].

##### Normalization

Image normalization is a pre-processing technique that scales pixel values to a consistent range. This helps improve the stability and performance of ML models. The pixel intensity values in an image typically range from 0 to 255 for 8-bit images. To normalize an image, these values are often rescaled to a range between 0 and 1, using the formula [28]:

(1) $$x^{\prime }=\frac{x-\min\left(x\right)}{\max\left(x\right)-\min\left(x\right)}$$

where *x* is the original pixel value, min (*x*) and max (*x*) are the minimum and maximum pixel intensities in the image, and $x^{\prime }$ is the normalized value. This transformation ensures that the pixel values are on a common scale, which can enhance the effectiveness of subsequent machine learning algorithms.

##### Standardization

Image standardization is a pre-processing step that transforms the pixel values of an image to have a mean of 0 and a standard deviation of 1. This process ensures that the data are centered around 0 and has a consistent scale. This transformation is crucial for many ML models, particularly DL algorithms, as it helps stabilize the learning process by reducing the variance between input features [29].

(2) $$x^{\prime }=\frac{x-\mu }{\sigma }$$

where *x* is the original pixel value, μ is the mean pixel value across the image, σ is the standard deviation, and $x^{\prime }$ is the standardized pixel value. By subtracting the mean μ and dividing it by the standard deviation σ, the standardization process ensures that the pixel values are distributed with a mean of 0 and a variance of 1. This is beneficial for neural network training, as it helps make the gradients more stable and reduces issues caused by varying pixel intensities across different images. This, in turn, allows the neural network to train more efficiently.

##### Noise Reduction

Noise reduction in images is an essential preprocessing step to remove unwanted random variations, known as noise, while preserving important features. Noise can originate from various sources, such as low light, sensor imperfections, or transmission errors, and can significantly degrade the quality of an image. A common technique for noise reduction is Gaussian filtering, which applies to a Gaussian kernel to smooth the image. The value of a pixel at position (*i*,*j*) after filtering is computed as follows [30]:

(3) $$I^{\prime }\left(i,j\right)=\sum_{m=-k}^{k}\sum_{n=-k}^{k}I\;\left(i+m,j+n\right)\cdot G\left(m,n\right)$$

where $I\left(i,j\right)$ is the original pixel value, *G* (*m*, *n*) is the Gaussian kernel, and *k* defines the kernel size. The Gaussian kernel is defined by

(4) $$G\left(x,y\right)=\frac{1}{2\pi \sigma^{2}}\;e^{-\frac{x^{2}+y^{2}\;}{2\sigma^{2}}}$$

where σ is the standard deviation of the Gaussian distribution, controlling the degree of smoothing. Another method is median filtering, where the pixel value is replaced with the median value of the surrounding pixels within a window. This is especially effective in removing “salt and pepper” noise, where random pixels are set to extreme values. Noise reduction improves the overall quality of an image, making it more suitable for further processing or analysis.

#### 3.2.2. Deep Feature Extraction

CNNs are DL models mainly utilized for image recognition and computer vision tasks. These models are engineered to autonomously and adaptively understand spatial hierarchies of features present in input images. A CNN comprises several layers, each dedicated to a particular function of feature extraction and classification [31]. The first layer of the model processes the input image, often visualized as a 2D array of pixel values. For this scenario, the input image would be a facial photograph of the evaluated person. In this layer, the image’s dimensions are adjusted to align with the model’s specific needs. The convolutional layer serves as the essential component of a CNN [32]. CNNs utilize various filters, often kernels, that traverse the input image to perform convolution operations. These filters are designed to detect specific patterns, including edges, textures, and more complex features in the image. Through training on extensive datasets, the filters learn to identify these characteristics. In image recognition, convolutional layers play a crucial role in feature extraction. The early layers of a CNN concentrate on capturing fundamental features such as edges and lines. These essential features are subsequently passed on to deeper layers responsible for recognizing more complex attributes, including facial expressions and tracking eye movements.

Key Parameters of a Convolutional Layer are as follows:

1. **Filters/Kernels**: These small windows slide over the input image, learning to recognize specific features like edges, corners, and textures. Each filter serves as a feature detector, and during the training process, the network fine-tunes the filter’s parameters to capture significant patterns.
2. **Stride**: This parameter determines the step size at which the filters move across the input image horizontally and vertically. A larger stride can decrease the spatial dimensions of the output feature maps, enhancing computational efficiency but potentially sacrificing some details. Conversely, smaller strides yield more detailed feature maps.
3. **Padding**: This involves adding extra pixels around the input image, which assists the filters in effectively processing the edges and border regions. Padding helps maintain the spatial dimensions of the feature maps, which is crucial for preventing information loss at the image’s edges, as shown in Equation (2).

After the convolution operation, a Rectified Linear Unit (ReLU) activation function is applied to the convolved output on an element-wise basis. This step introduces non-linearity into the model, allowing it to capture more intricate relationships between features. ReLU permits positive values to pass through while converting negative values to zero. [33].

(5) $$y\left(t\right)=\left(x*f\right)\left(t\right)=\int x\;\left(a\right)f\left(t-a\right)da$$

where the filter is represented by *f*(*t*), while *x*(*t*) and *y*(*t*) denote the input and output, respectively.

The main goal of ViT, DenseNet121, MobileNet, ResNet101V2, Xception, and InceptionV3 is to extract and represent image features as feature vectors. This was achieved by removing the classification layers and keeping the convolutional, pooling, and auxiliary layers. A global average pooling (GAP) layer was then used to transform the high-level feature maps into compact feature representations. The GAP operation simplified the spatial data by reducing each feature map to a single value.

#### 3.2.3. PCA

PCA is a statistical technique that transforms a large dataset with potentially correlated variables into a smaller set of uncorrelated variables called principal components. These components are linear combinations of the original variables chosen to maximize the variance of the data. PCA works as the following steps [34]:

1. **Standardization**: Since PCA is affected by the scale of variables, it is common to standardize the data to give all features equal importance. This is achieved by converting each feature to have a mean of 0 and a standard deviation of 1 (z-scores).
2. **Covariance Matrix Computation**: PCA computes the covariance matrix once the data are standardized to understand the relationships between different features. The covariance matrix shows how much the features vary together. This matrix helps determine how features are correlated with one another.
3. **Eigenvectors and Eigenvalues**: The covariance matrix is then decomposed into eigenvectors and eigenvalues. The eigenvectors represent the directions (principal components) of maximum variance in the data, while the eigenvalues represent those directions’ magnitude (variance).
4. **Choosing Principal Components**: Typically, only a few principal components are retained to reduce the dimensionality of the data. The number of components to retain is often chosen by looking at the explained variance ratio, which shows the proportion of total variance explained by each principal component.
5. **Projection onto Principal Components**: The original data are then projected onto the selected principal components to obtain a new, lower-dimensional data representation. This projection reduces the complexity of the dataset while preserving the most important patterns and relationships.

Our approach utilized PCA following feature extraction from the pre-trained DL models, including ViT, DenseNet121, MobileNet, ResNet101V2, Xception, and InceptionV3. Specifically, we extracted features from the penultimate layer of these six models to serve as input for PCA. The features transformed by PCA were then used as input for ML classifiers, namely RF, XGBoost XGB, AdaBoost, DT, and SVM, for classification tasks. PCA is crucial in selecting the most significant features while reducing the dimensionality of the CXR features extracted by the aforementioned models for TB classification. Although these models effectively extract meaningful features from images, the resulting features are often high-dimensional. They may contain redundancy, leading to increased computation time and potential noise or irrelevant information in the classification process. By applying PCA, we successfully reduced the dimensionality of the extracted features while preserving the essential information. This enabled us to concentrate on the most relevant features for TB detection.

Applying PCA to features extracted from CXR images enabled 2D or 3D visualizations, revealing key feature clusters and relationships. This provided insights into patterns and variations in the data, aiding in understanding features associated with TB.

PCA compressed extracted features into a lower-dimensional representation, reducing memory and computational demands during classification. By selecting the most critical features, PCA provided more informative input for machine learning models such as RF, XGB, DT, SVM, and AdaBoost, improving their generalization and performance in TB classification. Reducing dimensionality also minimized the risk of overfitting, enabling the models to perform better on unseen data.

The number of features extracted from DenseNet121, MobileNet, ResNet101V2, Xception, and Inception was 256, while the number of features extracted from ViT was 64. After applying PCA, the number of extracted features from ViT, DenseNet121, MobileNet, ResNet101V2, Xception, and Inception was reduced to 41.

#### 3.2.4. Vision Transformer

**Figure 3:**
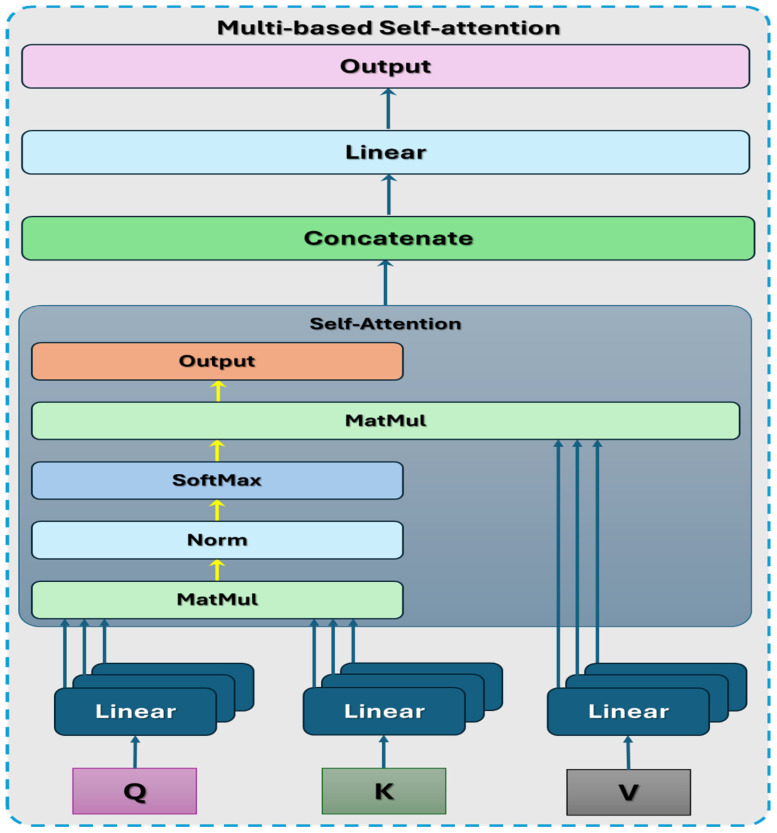
The multi-head self-attention process.

**Figure 4:**
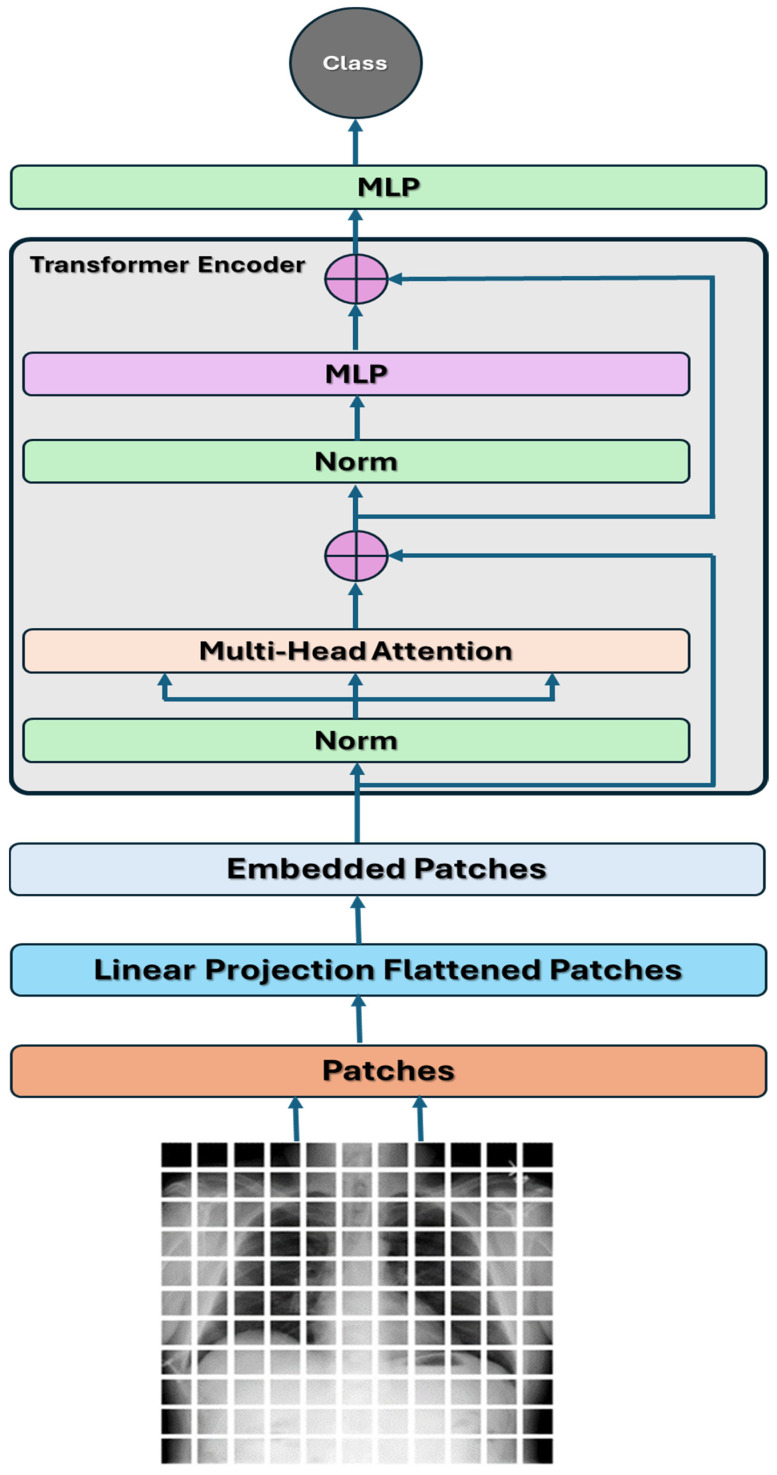
The ViT architecture.

ViT is a DL architecture introduced by Dosovitskiy et al. [35]. ViT applies the transformer architecture, initially developed for natural language processing (NLP), to image recognition. It represents a departure from traditional CNNs by leveraging self-attention mechanisms to process images. Self-attention allows the model to assess the significance of various sections of the input image during the prediction process. This mechanism is executed via an attention system, which computes a sum weighted for the input values, considering their associations with a specific query or position. The multi-head self-attention process is depicted in Figure 3. The components of the ViT architecture are depicted in Figure 4 and listed below [36]:

1. **Patch Embeddings**: The image provided is segmented into patches of a set size, with each patch undergoing a linear transformation to become a lower-dimensional embedding. These embeddings from the patches then function as the input for the transformer encoder.
2. **Transformer Encoder**: The transformer encoder comprises many self-attention mechanisms and multi-layer perceptron (MLP) layers. It handles the patch embeddings simultaneously, enabling the model to gather global context details from the whole image.
3. **Positional Encodings**: Since transformers do not naturally comprehend the spatial connections between patches, positional encodings are included in the patch embeddings. These encodings offer details about the relative positions of patches in the image.
4. **Classification Head**: After processing the patch embeddings through the transformer encoder, a classification head is typically added to the model to generate predictions for various tasks like image classification, object detection, or segmentation.

Compared to traditional CNN-based approaches, ViT has the following advantages [36]:

1. **Global Context**: Transformers can understand far-reaching connections and interactions among various sections of an image. This capability enables them to thoroughly examine the entire image when making predictions.
2. **Flexibility**: ViTs are versatile and can be used for tasks beyond just image classification, such as object detection, segmentation, and image generation.
3. **Efficiency**: ViTs can achieve greater memory efficiency than CNNs by independently processing image patches, particularly for large images.

The mathematical model for the ViT can be described as follows:

1. **Input Embedding**: When presented with an input image I, it gets split into patches of a set size. Every individual patch *P_i_* is later transformed using a trainable linear projection matrix *W_e_* to have a reduced dimensionality in an embedding space. Let *x_i_* represents the embedding of a patch *P_i_*, then, the input embeddings can be represented as follows:
  (6) $$x_{i}=W_{e}\;\cdot \;P_{i}$$
2. **Transformer Encoder**: The input embeddings, in combination with the positional encodings, are inputted into a series of transformer encoder layers. Each encoder layer is comprised of two key parts:
  - **Multi-Head Self-Attention**: This process lets the model focus on various sections of the embedding input simultaneously. When provided with input embeddings *X*, the multi-head self-attention output is determined as
    (7) $$Attension\left(Q,K,V\right)=softmax\left(\frac{QK^{T}}{\sqrt{d_{k}}}\right)\;V$$
    where $Q=XW_{Q},\;K=XW_{k},\;\mathrm{and}\;V=XW_{v}$ are linear projections of the input embeddings and $W_{Q},\;W_{k},\;\mathrm{and}\;W_{v}$ are learnable linear projection matrices. $d_{k}$ represents the dimensionality of the key vectors.
  - **Feedforward Neural Network**: The self-attention process results through a feedforward neural network (FFNN) made up of two linear changes divided by a non-linear activation function (usually ReLU). The FFNN result is determined in a manner that it can be described as
    (8) $$FFNN\left(x\right)=ReLU\left(xW_{1}+b_{1}\right)W_{2\;}+b_{2}$$
    where $W_{1}$ and $W_{2}$ are learnable weight matrices and $b_{1}$ and $b_{2}$ are bias vectors.
  - **Classification Head:** The transformer encoder’s output embeddings are usually combined into one representation, for example, using average pooling. This combined representation then goes through a linear layer and a softmax activation function to produce probabilities for different classes in image classification tasks.

In the proposed ViT model, an image is initially received with 3 RGB channels and a resolution of 224 × 224 pixels. The process commences with the patch layer, which dissects the input image into smaller chunks known as “patches”. Each patch encapsulates a distinct segment of the image. The ViT architecture executes a transformation on each patch. The transformations change the segments of images into a collection of vector representations, which are subsequently handled within the ViT model’s encoder blocks.

An encoder block of the ViT model receives data from (None, 121, and 64) and produces a result in the same (None, 121, and 64). These blocks consist of three main components:

1. An attention mechanism.
2. An intermediate layer.
3. A final layer.

**Figure 5:**
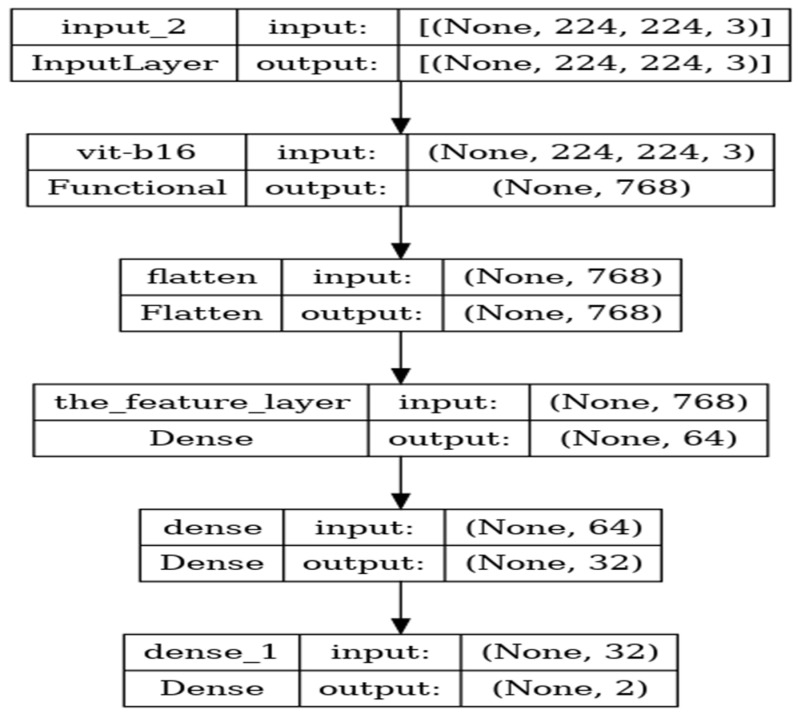
The proposed ViT model’s architecture.

In the ViT model, the attention component captures image features and highlights essential details. The intermediate layer plays a vital role in enhancing the features identified by the attention mechanism, making them more complex and comprehensive. This intermediate layer includes the dense and dropout layers and is supported by the GELU activation function. In the final phase, the output layer reverts the processed characteristics to their original sizes while utilizing a Dropout layer to introduce variety and prevent overfitting. Including LayerNormalization is essential for both before and after the encoding phase in each block. This method enhances the stability and robustness of the model through the normalization of the output and the application of weighted normalization processes. After the 12th encoder block, the normalization layer normalizes the data, initially with dimensions of (None,1024), to generate an output of dimensions (None, 2). This methodology empowers the model to make predictions for TB from CXR images. The proposed ViT model’s architecture is shown in Figure 5:

The proposed ViT model consisted of 12 transformer encoder layers, each featuring 16 attention heads. The configuration of this model is illustrated in Figure 5. The first layer, ViT-B16, is a custom Keras layer that implements the ViT-B16 architecture, outputting a 768-dimensional vector. Following this, the flattening layer converts the 768-dimensional vector into a 1-dimensional vector, essential for input preparation for the subsequent batch normalization layer. The batch normalization layer normalizes the values of the input vector. The next component, a dense layer, is fully connected and comprises 64 output units. This layer is tasked with learning to classify the 64 types of TB. Afterward, a batch normalization layer normalizes the output from this dense layer. The final layer is another dense layer comprising two output units, which generate the predicted class labels for the input image. Overall, the ViT-b16 model contains a total of 85,810,279 parameters, which include 85,808,721 trainable parameters and 1558 non-trainable parameters.

## 4. Results and Discussion

### 4.1. Experimental Setup

We used the Kaggle environment to implement the proposed fine-tuned CAD model. The used hyperparameter values for the proposed fine-tuned CAD system are shown in Table 3. Kaggle is a well-known online platform for data science competitions. Researchers can engage in challenges aimed at solving real-world issues by applying data analysis and machine learning methods. Established in 2010, Kaggle offers space for data scientists, ML engineers, and researchers to work together, exchange datasets, analyze data, and create predictive models.

Our research integrated ViT with PCA and ML techniques to classify TB. In the proposed hybrid CAD system, ViT was used to extract deep features, PCA was utilized to minimize the dimensionality of these features, and different ML methods were implemented to classify TB. During the pre-processing phase, the input CXR images were modified to fit the specifications of the ViT model. Since the standard input size for the pre-trained ViT model is 224 × 224 pixels, all images in the dataset were resized accordingly. The ML models were trained using the AdamW optimizer on the designated training samples from the TB Chest X-ray dataset. Throughout the training process, adjustments were made to the pre-existing weights of the ML models to improve their performance on the TB Chest X-ray dataset. The batch size was 256, and the learning rate was fixed at 0.0001. Although the maximum number of epochs was capped at 100, an early stopping mechanism was implemented to monitor the validation loss and appropriately halt the training when necessary.

### 4.2. Evaluation Metrics

In this research, we evaluated the proposed fine-tuned CAD system using precision, recall, F1-score, AUC, accuracy, and specificity:

- **Precision** focuses on the quality of positive predictions made by the model. A high precision value (closer to 1) indicates that most of the positive predictions made by the model were correct. A low precision value indicates that the model makes many false positive predictions.
- **Recall**, also known as the True Positive Rate (TPR), measures the completeness of positive predictions made by the model. A high recall value (closer to 1) indicates that the model accurately identifies a large proportion of all actual positive cases. Conversely, a low recall value suggests the model fails to detect many true positive cases, leading to false negatives.
- **F1-score**, also known as F-measure or balanced F-score. It is the harmonic means of precision and recall. It addresses a key limitation of using only precision or recall individually by combining them into a single score. F1-score ranges from 0 to 1, with 1 indicating perfect performance (both precision and recall are 1). A high F1-score (closer to 1) suggests the model identifies true positives and minimizes false positives/negatives. A low F1-score indicates a significant imbalance towards either high precision or high recall, requiring further analysis.
- **Specificity**, also known as the true negative rate (TNR), focuses on correctly identifying negative instances. A high specificity value (close to 1) ensures the model can effectively identify many negative instances. On the other hand, a low specificity value ensures that the model mistakenly labels many true negative instances, resulting in false positives.
- **Accuracy** provides a general overview of how often the model makes correct predictions, regardless of the specific class. A high accuracy value (closer to 1) suggests the model performs well overall in making correct predictions. A low accuracy value indicates that the model makes many incorrect predictions.
- **False Negative Rate (FNR)**, also known as the false omission rate, is a metric used to evaluate the performance of a binary classification model. It measures the proportion of actual positive cases incorrectly classified as negative by the model. In other words, it represents the rate at which the model fails to identify positive instances when they exist.
- **Negative Predictive Value (NPV)** is a statistical measure used to assess the performance of a diagnostic test or model, particularly in the context of binary classification problems. It represents the proportion of true negative predictions among all the cases predicted as negative by the model [37,38].

Accuracy = (TP + TN)/(TP + TN + FP + FN) (9)

Precision = TP/(TP + FP) (10)

Recall = TP/(TP + FN) (11)

Specificity = TN/(TN + FP) (12)

F1-score = (2 × recall × precision)/(recall + precision) (13)

FNR = FN/(TP + FN) (14)

NPV = TN/(TN + FN) (15)

### 4.3. The Proposed CAD Model Evaluation

In our experiments, we carried out a binary classification task using CXR samples from the TB Chest X-ray dataset containing two distinct classes: TB and normal. We started by preparing the CXR images from this dataset. This preparation included resizing, scaling, and removing noise from the images. We divided the dataset into two parts: 80% of the images, totaling 3360 CXR samples, were used for training, while the remaining 20%, or 840 CXR samples, were reserved for testing.

We conducted two experiments. In the first experiment, we used transfer learning to pre-train the six DL models. The initial phase involved supervised pre-training on the ImageNet dataset. After that, we fine-tuned the six DL models using the training data from the TB Chest X-ray dataset. We then evaluated various metrics, as described in Equations (9)–(15), across these six DL models as classifiers.

We also utilized transfer learning in the second experiment to pre-train the six DL models. The first step was supervised pre-training with the ImageNet dataset to extract deep features from the CXR images. Next, we fine-tuned the five ML models with each of the six DL models using the training data from the TB Chest X-ray dataset. We assessed various metrics, as detailed in Equations (9)–(15), across the five ML models as classifiers. During this second experiment, we employed each one of the six DL models as a base model to extract deep features, used PCA to reduce the dimensions of these features, and applied several ML methods (RF, XGB, DT, SVM, and AdaBoost) to classify the TB cases.

In the first experiments, the outcomes produced by the ViT, DenseNet121, MobileNet, ResNet101V2, Xception, and InceptionV3 as classifiers can be observed in Table 4, Table 5, Table 6, Table 7, Table 8 and Table 9. The average evaluation measures for the six DL models in the binary classification task on the TB Chest X-ray dataset BT dataset are presented in Table 4, Table 5, Table 6, Table 7, Table 8 and Table 9. The ViT model achieved the highest accuracy of 99.4%, followed by DenseNet121 at 99.29%, InceptionV3 at 98.815, Xception at 98.33%, MobileNet at 97.98%, and ResNet101V2 at 97.38%. Therefore, it can be concluded that the ViT model performed the most accurately among the other DL models analyzed.

The ViT achieved an average accuracy of 99.4%, which indicates the percentage of correct predictions made. It also has a specificity of 98.765%, representing the proportion of actual negatives that were correctly identified. The FNR is 1.235%, indicating the percentage of actual positives that were incorrectly classified. Additionally, NPV is 99.05%. The model correctly predicted the absence of a condition, with a precision of 99.05%, recall of 98.765%, and 98.905% F1-score. DenseNet121 got an average accuracy of 99.29%. Its specificity rate was 98.4%. It had a FNR of 1.6% and a NPV of 98.975%. The precision and recall rates were 98.975% and 98.4%, respectively. Additionally, it had an F1-score of 98.68%. MobileNet recorded an average of accuracy 97.98%, specificity 94.97%, FNR 5.03%, NPV 97.525%, precision 97.525%, recall 94.97%, and 96.19% F1-score. ResNet101V2 recorded an average accuracy of 97.38%, specificity of 96.965%, FNR of 3.035%, NPV of 93.935%, precision of 93.935%, recall 96.965%, and 95.365% F1-score. Xception achieved an accuracy rate of 98.33%, specificity rate of 95.48%, FNR stood at 4.52%, NPV of 98.375%, precision score also reached 98.375, recall, at 95.48%, and F1-score, at 96.855%.

InceptionV3 attained an accuracy rate of 98.81%, a specificity rate of 97.82%, a FNR of 2.18%, a NPV of 97.82%, a precision score matching the NPV at 97.82%, a recall rate of 97.82%, and an F1-score of 97.82%.

Therefore, ViT showcased exceptional performance across various evaluation metrics. It achieved the highest average values for accuracy (99.4%), specificity (98.765%), NPV (99.05%), precision (99.05%), recall (98.765%), and F1-score (98.905%). Additionally, it demonstrated the lowest average FNR at 1.235%. These results indicate that ViT excels in multiple aspects of the evaluation process, establishing it as a robust performer in the context of binary classification tasks.

In the normal class, ViT achieved the top scores for accuracy, specificity, NPV, recall, precision, and F1-score, with percentages of 99.4%, 97.81%, 98.53%, 99.72%, 99.57%, and 99.64, respectively. Among the models, DenseNet121 and Xception had the highest recall rate at 99.72%. ViT, DenseNet121, and Xception notably showed the lowest FNR at 0.28%.

For the class TB, the ViT model achieved the top scores for accuracy, specificity, NPV, precision, recall, and F1-score, with values of 99.4%, 99.72%, 2.19%, 99.57%, 98.53%, 97.81%, and 98.17%, respectively. DenseNet121 and Xception models excelled in specificity with a high score of 99.72%. ViT demonstrated the lowest FNR at 2.19%.

**Figure 6:**
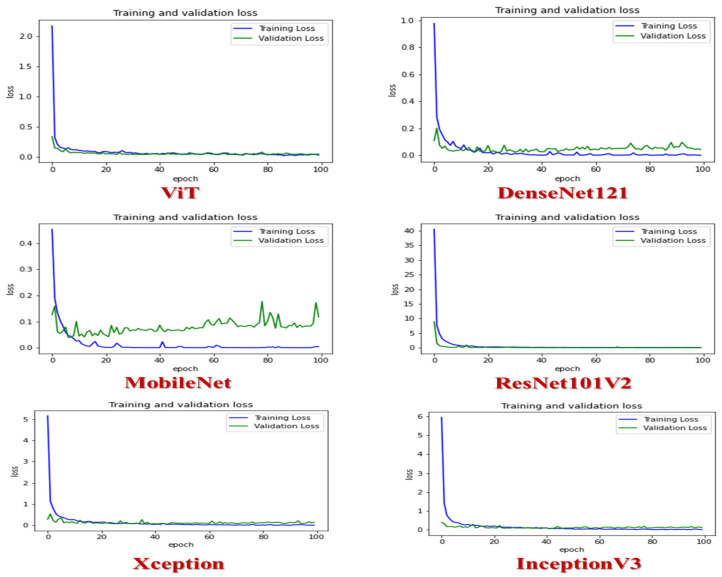
The training and validation loss for the Six CNNs.

Figure 6 presents the validation and training loss for the six distinct CNN models: ViT, DenseNet121, MobileNet, ResNet101V2, Xception, and InceptionV3. Among these models, ViT, DenseNet121, ResNet101V2, InceptionV3, and Xception displayed consistent performance without bias or variance. Both models exhibited decreased training and validation losses as the number of epochs increased. On the other hand, MobileNet demonstrated unstable performance due to the variance of the validation loss.

**Figure 7:**
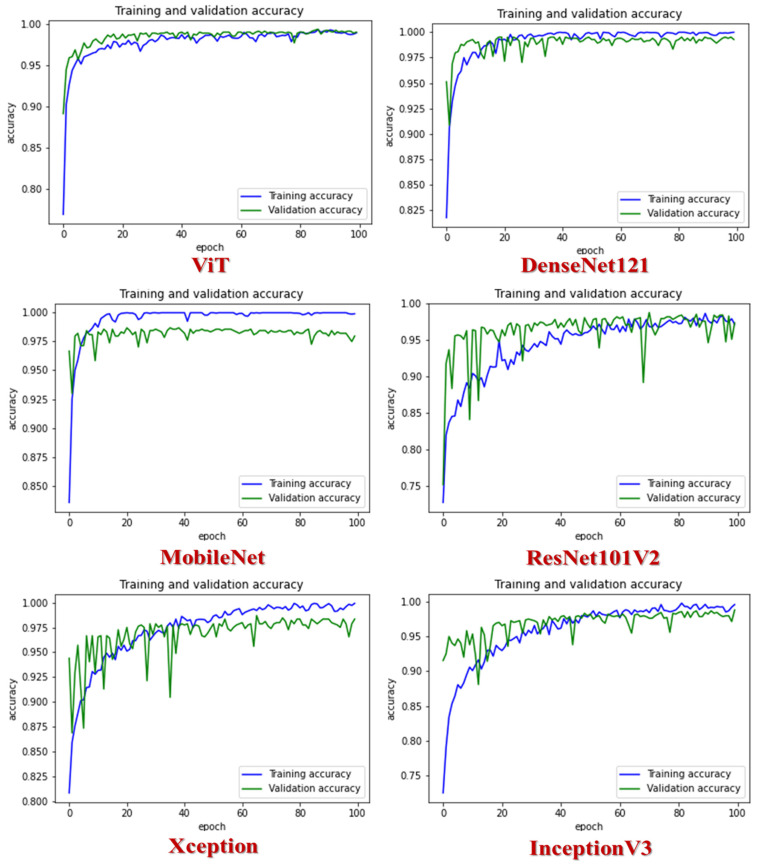
The accuracy of the six CNNs for the binary classification.

In Figure 7, we can observe the performance of the six different CNN models: ViT, DenseNet121, MobileNet, ResNet101V2, Xception, and InceptionV3. ViT, DenseNet121, and InceptionV3 demonstrated exceptional training and validation accuracy, reaching approximately 99% after the 80th epoch. MobileNet, on the other hand, achieved a training accuracy of over 97% at the 20th epoch, while its validation accuracy stood at 97.5%. ResNet-101V2 maintained a consistent performance, securing a training and validation accuracy level of around 97% after the 80th epoch. Xception also delivered a commendable performance, with a training accuracy of approximately 98%, while its validation accuracy stood at 97% after the 80th epoch.

**Figure 8:**
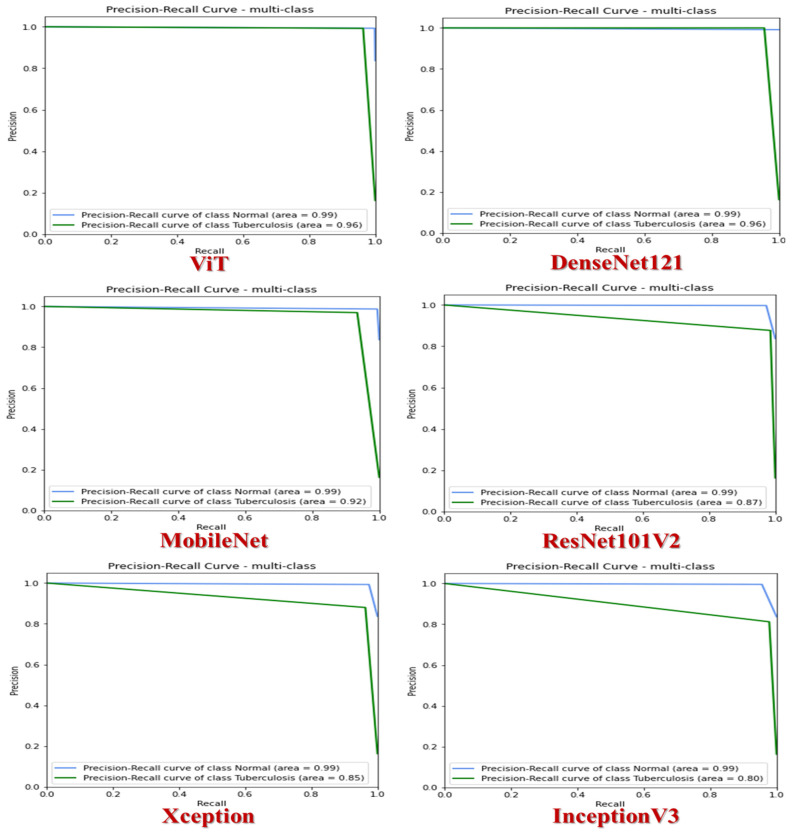
The precision–recall curves for the six CNN models for the binary classification.

Figure 8 shows the precision–recall curves for different CNN models—ViT, DenseNet121, MobileNet, ResNet101V2, Xception, and InceptionV3—applied to classify CXR samples from the TB Chest X-ray dataset. These curves demonstrate the trade-off between precision (*y*-axis) and recall (*x*-axis). Each curve depicts how well the model balances precision and recall at various decision thresholds. Focusing specifically on the ViT and DenseNet121 models, the curves for the normal class (blue curves) display an impressive AUC value of 99%, indicating strong performance. These curves closely follow the top-right corner of the graph, signaling high precision and recall levels across different thresholds.

On the other hand, the curves for the TB class (green curves) also exhibit a high AUC of 96%, highlighting the model’s effectiveness. Although these curves stay near the upper-right corner, they show a slightly more noticeable decline than the normal class curves. These high AUC values suggest that the model effectively distinguishes between the normal and TB classes. A higher precision at a certain recall level implies fewer false positives, while increased recall at a specific precision level suggests fewer false negatives. The sharp decrease towards the end of the curves indicates reduced precision as recall approaches 1 in some threshold cases, which may result in more false positives. Analyzing the ViT and DenseNet121 model’s precision–recall curves reveals their proficiency in differentiating between normal and TB cases, with slightly better performance in classifying normal cases.

In assessing the MobileNet model, the blue curve demonstrates outstanding performance in the normal category, achieving an impressive AUC of 99%. This signifies a high level of accuracy in classifying tasks. The curve consistently stays near the top-right corner, indicating strong precision and recall capabilities across various thresholds. Conversely, the green curve illustrates the performance in the TB category, achieving an AUC of 92%. While still robust, this performance is lower compared to the normal class. This curve shows a more significant decline in performance as recall values increase when contrasted with the normal class. The remarkable AUC value of 99% for the Normal category suggests nearly flawless classification performance, showcasing the model’s reliability in identifying normal cases.

On the other hand, though decent, the AUC value of 92% for the Tuberculosis category p, though decent points to a slight drop in performance, highlighting Moreover, the decrease in precision for the Tuberculosis category as recall nears 1 indicates an uptick in false positives at higher recall levels. Compared to previous models, MobileNet exhibits slightly lower performance for the Tuberculosis category (AUC of 92% versus 96% in the ViT and DenseNet121 models). To sum up, the precision–recall curves for MobileNet demonstrate exceptional performance for the normal class and good, though not perfect, performance for the TB category.

The ResNet101V2 model is highly effective at distinguishing between normal and TB categories based on two distinct curves. The blue represents the normal category and demonstrates exceptional accuracy with an AUC of 99%. This signifies the model’s remarkable precision in classification tasks, consistently placing it near the upper-right corner to signify strong precision and recall capabilities across various thresholds. On the other hand, the green curve reflects the model’s performance in the TB category, achieving a slightly lower AUC of 87% compared to the normal class. Unlike the stable performance of the normal curve, the TB curve shows a more significant decline as recall values increase. While the model excels in accurately classifying normal cases with a 99% AUC, there is room for improvement in detecting tuberculosis cases, as indicated by the 87% AUC. Furthermore, recall values near 1 for the TB category show a visible reduction in precision, suggesting an increase in false positives at higher recall levels. This underscores areas that can be enhanced to improve the detection of tuberculosis cases.

The Xception model effectively distinguishes between normal and TB cases by displaying two distinct curves. The blue curve represents the normal category with impressive accuracy, boasting an AUC of 99%. This underscores the model’s exceptional ability to accurately classify data, consistently positioning them in the upper-right corner and showcasing strong precision and recall capabilities across various thresholds. In contrast, the green curve indicates the model’s performance in identifying TB cases, achieving a slightly lower AUC of 85% compared to the normal category. Unlike the steady performance of the normal curve, the Tuberculosis curve shows a significant decline as recall values increase. While the model excels in identifying normal cases with a 99% AUC, there is room for improvement in detecting Tuberculosis cases, as evidenced by the 85% AUC. Furthermore, recall values near 1 for the TB category show a noticeable drop in precision, indicating an increase in false positives at higher recall levels. This highlights areas that can be enhanced to improve the accuracy of TB case identification.

The InceptionV3 model has shown impressive effectiveness in distinguishing between normal and TB cases by visualizing two separate curves. The blue curve represents the normal category with exceptional precision, achieving a high AUC of 99%. This showcases the model’s ability to accurately categorize data, consistently placing them in the top-right section and demonstrating strong precision and recall capabilities across various thresholds. On the other hand, the green curve illustrates the model’s performance in identifying TB cases, achieving a slightly lower AUC of 80% compared to the normal category. Unlike the stable performance of the normal curve, the TB curve shows a noticeable decline as recall values increase. While the model performs exceptionally well in detecting normal cases with a 99% AUC, there is room for improvement in identifying tuberculosis cases, evident from the 80% AUC. Furthermore, recall values near 1 for the TB category show a visible decrease in precision, indicating an increase in false positives at higher recall levels. This highlights areas for enhancements to enhance the accuracy of TB case detection.

**Figure 9:**
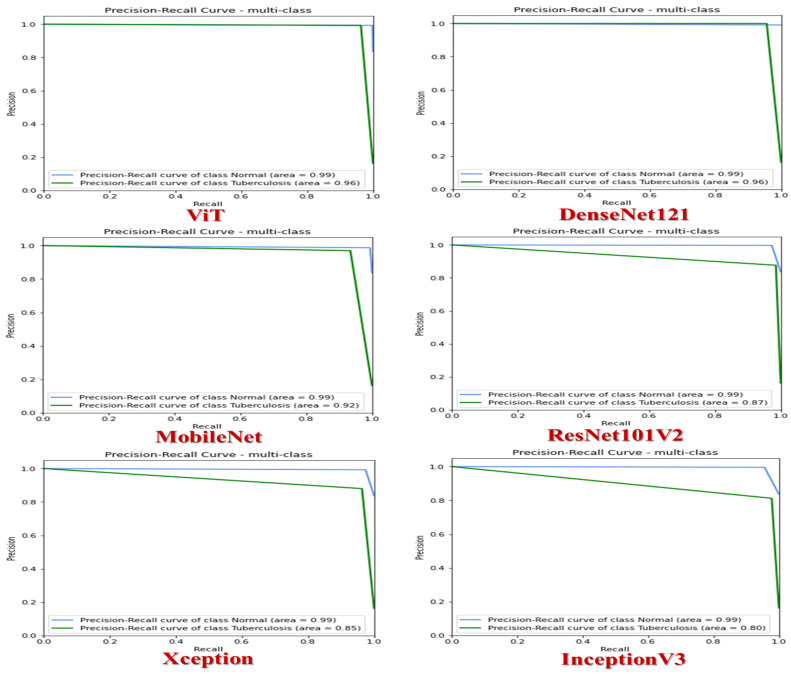
The ROC curves for the six CNN models for the binary classification.

Figure 9 displays the performance of six distinct CNN models (ViT, DenseNet121, MobileNet, ResNet101V2, Xception, and InceptionV3) in identifying TB in CXR samples from the TB Chest X-ray dataset. The evaluation was based on ROC curves, plotting the true positive rate (TPR) against the false positive rate (FPR). TPR represents the correctly identified positive cases, while FPR indicates the incorrectly identified negative cases. The analysis includes the AUC, assessing the models’ ability to differentiate between normal and TB classes. An AUC of 1 signifies a perfect model, while an AUC of 0.5 represents performance equivalent to random guessing. The ViT and DenseNet121 models exhibited exceptional performance with a micro-average ROC curve of 99%, demonstrating strong performance across all classes. Furthermore, the ROC curves for both normal and TB classes individually displayed high AUC values of 98%, indicating the model’s efficacy in distinguishing between healthy individuals and those with Tuberculosis. In conclusion, the analysis of the ROC curves underscores the model’s effectiveness in accurately identifying patients with Tuberculosis from CXR images.

When assessing the performance of the MobileNet model, we find impressive outcomes in the analysis of the ROC curve. The micro-average ROC curve demonstrates strong performance, achieving a rate of 99%. Similarly, the ROC curves for the normal and TB classes exhibit effectiveness at 96% each. The micro-average ROC curve offers a comprehensive view of the model’s performance across all classes. The AUC value of 99% signifies that the model distinguishes between healthy individuals and those with either disease. However, the individual ROC curves for the Normal and Tuberculosis classes show slightly lower AUC values of 96%. This disparity suggests that the model’s accuracy in distinguishing these specific classes is marginally lower than its overall performance.

Impressive results are seen when evaluating the ROC curve for the ResNet101V2 model. The average ROC curve, which combines all categories, shows strong effectiveness with a success rate of 98%. Specifically, the ROC curves for the normal and TB categories exhibit high efficiency, each reaching 98%. This average ROC curve gives a comprehensive view of the model’s effectiveness across all categories. With an AUC value of 98%, the model differentiates between individuals with and without diseases. Nonetheless, the individual ROC curves for the healthy and TB categories reveal slightly lower AUC values of 98%. This discrepancy suggests that the model’s accuracy in distinguishing these specific categories is slightly lower than its overall performance.

The evaluation of the ROC curve for the Xception model shows excellent results. The average ROC curve, covering all categories, demonstrates impressive efficacy with a success rate of 98%. Specifically, the ROC curves for the healthy and TB categories exhibit notable efficiency, each achieving a success rate of 97%. This comprehensive average ROC curve gives a complete overview of the model’s effectiveness across all categories. With an AUC value of 98%, the model accurately identifies individuals with and without diseases. However, the individual ROC curves for the Healthy and Tuberculosis categories show slightly lower AUC values of 97%. This difference suggests that the model’s accuracy in distinguishing these specific categories is slightly lower than its overall performance.

The assessment of the ROC curve results for the InceptionV3 model shows a high level of performance. The overall ROC curve, covering all classifications, demonstrates impressive effectiveness with a success rate of 97%. Specifically, the ROC curves for the healthy and TB categories exhibit significant efficiency, each achieving a success rate of 97%. This comprehensive ROC curve summarizes the model’s efficacy across all categories. With an AUC value of 97%, the model accurately identifies individuals with and without the respective diseases. However, the individual ROC curves for the healthy and TB categories show slightly lower AUC values of 97%. This difference suggests that the model’s precision in distinguishing these specific categories is somewhat reduced compared to its overall performance.

In the second experiment, we used each one of the six DL models as a based model to extract deep features. We then applied PCA to reduce the dimensionality of these features. We implemented several ML methods, including RF, XGB, DT, SVM, and AdaBoost, to classify the TB cases. The results from the deep feature extractors (base models)—ViT, DenseNet121, MobileNet, ResNet101V2, InceptionV3, and Xception—and the five ML models (RF, XGB, DT, SVM, and AdaBoost) as classifiers are detailed in Table 10, Table 11, Table 12, Table 13, Table 14, Table 15 and Table 16. The average evaluation measures for the RF, XGB, DT, SVM, and AdaBoost models in the binary classification task on the TB Chest X-ray dataset are also presented in Table 10, Table 11, Table 12, Table 13, Table 14, Table 15 and Table 16.

From Table 10, ViT demonstrated consistently high accuracy across all classifiers, achieving results between 99.68% and 99.84%. This indicates ViT’s effectiveness with most classifiers, particularly with RF, XGB, and AdaBoost, which reached the highest accuracy of 99.84%. ResNet50’s accuracy was lower overall than other models, particularly with DT at 88.25% and AdaBoost at 91.11%. SVM provided the best results for ResNet50 at 96.19%. DenseNet121 performed strongly across all classifiers, achieving the highest accuracy with SVM at 99.05% and XGB at 98.57%.

Nevertheless, DT again resulted in relatively lower accuracy at 95.24%. Xception exhibited moderate variance in performance depending on the classifier, with the highest accuracy from SVM at 97.94% and the lowest from DT at 90.63%. Like other models, Xception performed better with advanced classifiers such as SVM and XGB than DT. InceptionV3 showed similar performance patterns to Xception, with a highest accuracy of 97.46% using SVM and a lowest of 90.32% with DT. MobileNet was among the top-performing models, particularly with SVM at 99.05% and AdaBoost at 98.10%. Even DT provided a reasonable performance of 95.08% with MobileNet, suggesting it has broader compatibility with classifiers than other models. SVM consistently produced the highest or near-highest accuracy for most DL models, demonstrating its effectiveness in managing complex feature representations. In contrast, DT consistently showed lower performance across all models, indicating it may not be as effective for processing deep features from neural networks.

**Figure 10:**
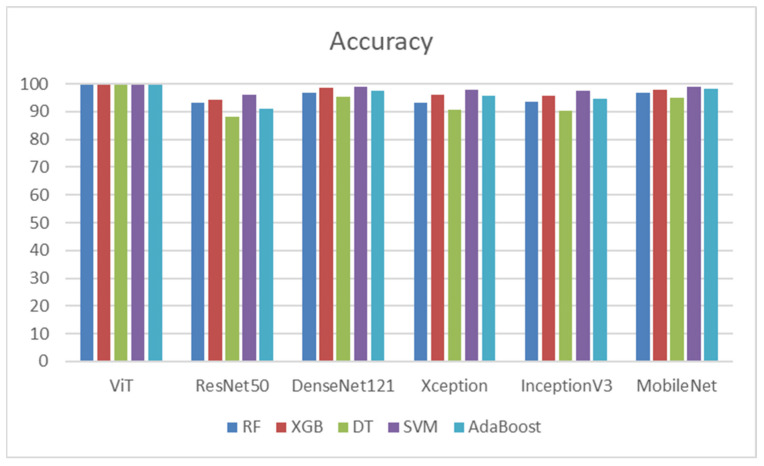
The accuracies of the five ML models.

In summary, ViT followed by DenseNet121, was identified as the best-performing DL model, particularly when used with classifiers such as SVM and XGB. AdaBoost and XGB showed strong performance, especially with MobileNet, ViT, and DenseNet121, highlighting their effectiveness in ensemble learning methods. ViT followed by DenseNet121 and MobileNet produced the most favorable outcomes for tasks that demand high accuracy when paired with advanced classifiers like SVM or XGB. Figure 10 shows the accuracies of the five ML models.

ViT demonstrated exceptional specificity across all classifiers, achieving a range from 99.05% with SVM to 99.52% with RF, XGB, and AdaBoost, as shown in Table 11. This highlights ViT’s effectiveness as a feature extractor, delivering outstanding results with most classifiers. ResNet50’s performance was comparatively lower than other models, particularly with DT (79.62%) and AdaBoost (79.81%). The highest specificity for ResNet50 was observed with SVM at 88.95%, suggesting it has potential when used with more sophisticated classifiers. DenseNet121 showcased a strong overall performance, particularly with SVM (97.52%) and XGB (96.48%). Although there was a slight decrease in specificity with DT (90.67%), DenseNet121 consistently ranked among the top-performing models.

**Figure 11:**
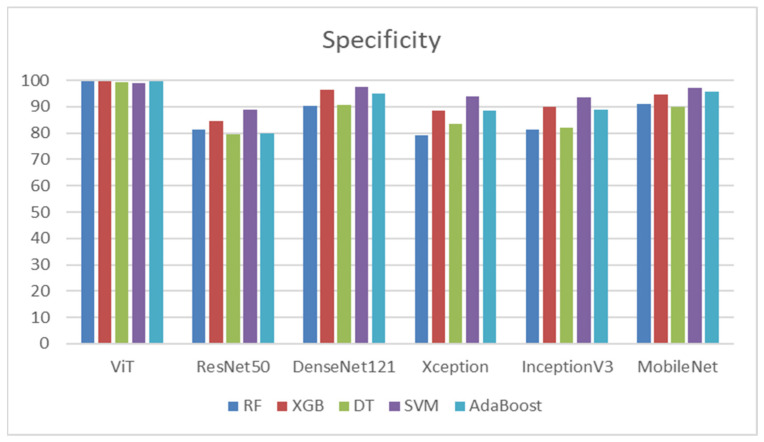
The specificity of the five ML models.

Xception achieved good results with SVM (93.81%) but showed lower performance with simpler classifiers like DT (83.33%). It also performed solidly with XGB (88.48%) and AdaBoost (88.57%), indicating that Xception pairs well with more advanced techniques. InceptionV3 exhibited a performance pattern similar to Xception, with the highest specificity recorded by SVM (93.52%) and the lowest by DT (82.00%). InceptionV3 also performed reasonably well with XGB (89.90%) and AdaBoost (88.86%). MobileNet achieved high specificity, particularly with SVM (97.14%) and AdaBoost (95.81%). Although DT yielded lower specificity (89.81%), it remained acceptable, making MobileNet a robust model overall. SVM emerged as the best-performing classifier, achieving the highest specificity for most DL models. In contrast, DT consistently produced lower specificity across all models, suggesting it may not be the most suitable classifier for managing complex features extracted by DL models. ViT and DenseNet121 are highlighted as the top feature extractors, achieving high specificity across all classifiers. XGB and AdaBoost also performed well, particularly with models like DenseNet121 and MobileNet, showcasing their strength in enhancing performance through ensemble methods. Figure 11 shows the specificities of the five ML models.

**Figure 12:**
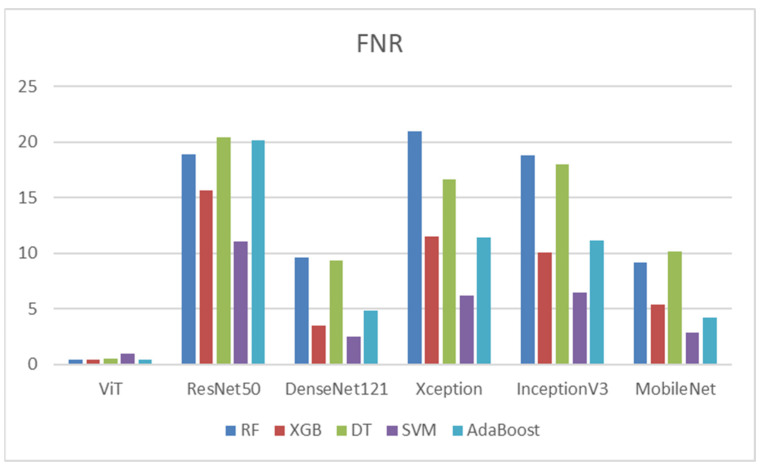
The FNRs of the five ML models.

Table 12 indicates that ViT recorded the highest FNR with SVM at 0.95. In comparison, RF, XGB, and AdaBoost exhibited the lowest FNR at 0.48. Xception exhibited the highest FNR of 20.95 with RF, while the lowest FNR was recorded at 6.19 with SVM. InceptionV3 performed poorly with RF, presenting the highest FNR of 18.76, whereas SVM performed better with a FNR of 6.48. Lastly, MobileNet achieved the highest FNR of 10.19 with DT, while its lowest FNR of 2.86 was observed with SVM. In conclusion, ViT combined with RF, XGB, or AdaBoost generally maintained the lowest FNR across most deep features, indicating that they tend to produce fewer FN compared to other classifiers. Conversely, RF combined with Xception and DT frequently exhibited the highest FNR, suggesting an increase in false negatives across different DL models. Figure 12 shows the FNRs of the five ML models.

**Figure 13:**
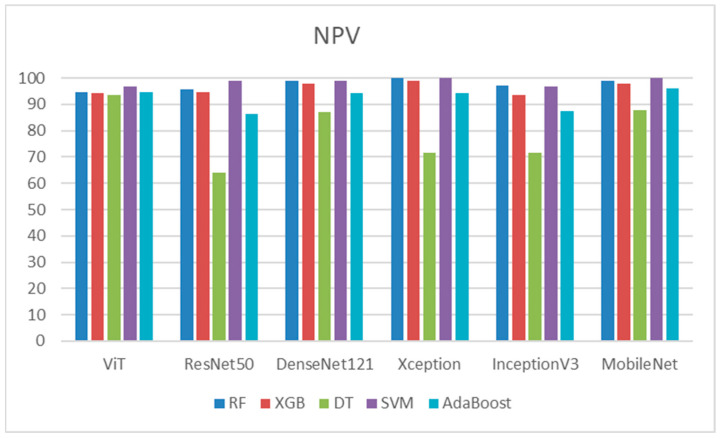
The NPVs of the five ML models.

Table 13 demonstrates that SVM consistently outperformed nearly all deep feature models. It achieved a perfect NPV of 100% for Xception and MobileNet features, with other models, such as DenseNet121, achieving near-perfect scores of 99%. Xception proved to be the most effective feature extractor for these classifiers, obtaining a perfect NPV of 100% using both SVM and RF. DenseNet121 also showed strong results, reaching 99% with SVM and nearly 98.8% with RF. ResNet50 displayed variability in performance; it excelled with SVM at 98.8% but faced challenges with DT, scoring only 64.2%. The ViT maintained bal-anced performance across classifiers, though none surpassed 96.8%. InceptionV3 per-formed well overall but lagged slightly behind other models, particularly with DT and AdaBoost. MobileNet delivered high performance, often matching or nearing the top scores for most classifiers. However, DT struggled significantly with all deep feature mod-els, especially with ResNet50 (64.2%) and Xception (71.7%). AdaBoost showed good per-formance with MobileNet (96%) and DenseNet121 (94.1%) but was relatively weaker with ResNet50 and InceptionV3. Figure 13 shows the NPVs of the five ML models.

**Figure 14:**
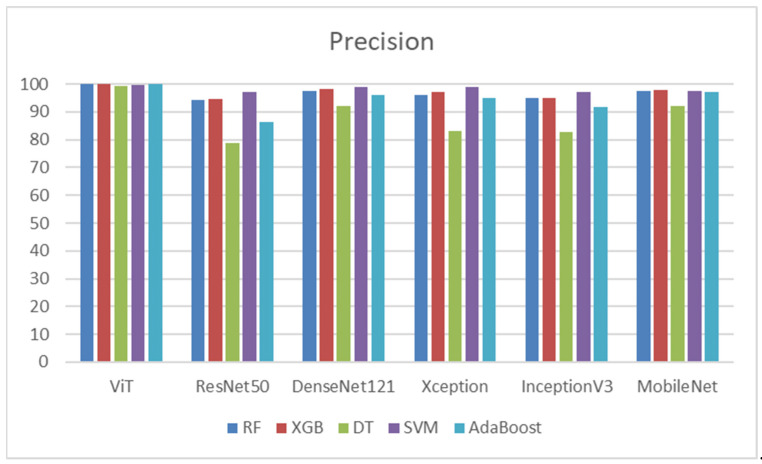
The precisions of the five ML models.

As shown in Table 14, ViT and all classifiers achieved high precision, with RF, XGB, and AdaBoost reaching 99.90%. SVM was followed closely, with a precision of 99.81%. DT had a slightly lower precision of 99.43%. ResNet50 combined with SVM showed the highest precision at 97.30%, compared to DT’s lowest precision of 78.75%. DenseNet121 with SVM again outperformed other classifiers with a precision of 99.03%, while DT was lower at 91.96%. Xception paired with SVM achieved a precision of 98.79%, while DT’s precision was significantly lower at 83.08%. InceptionV3 combined with SVM reached the highest precision at 97.21%, while DT lagged behind at 82.75%. MobileNet, SVM, and RF also displayed high precision at 97.68%, while DT had the lowest precision at 92.09%. SVM consistently delivered the highest precision across most DL models, indicating its effectiveness in accurately predicting positive samples. In contrast, DT consistently demonstrated the lowest precision across the models, suggesting challenges in correctly classifying positive samples compared to other classifiers. Figure 14 shows the precisions of the five ML models.

**Figure 15:**
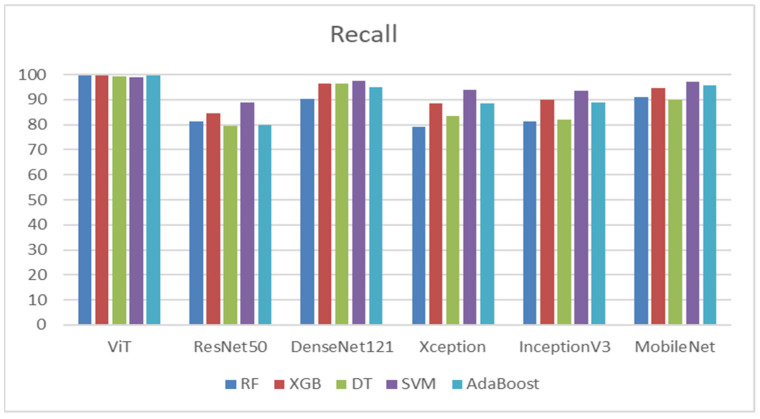
The recalls of the five ML models.

Table 15 depicts that the ViT model demonstrated the best recall among all classifiers, particularly with RF, XGB, and AdaBoost. DenseNet121 also performed well, especially with SVM, achieving a recall of 97.52%. ResNet50 had the weakest overall performance, particularly with DT and AdaBoost. MobileNet exhibited fairly strong performances with SVM. These results indicate that ViT and DenseNet121 were consistently reliable across classifiers for achieving high recall, while ResNet50 generally underperformed compared to the other models. Figure 15 shows the recalls of the five ML models.

**Figure 16:**
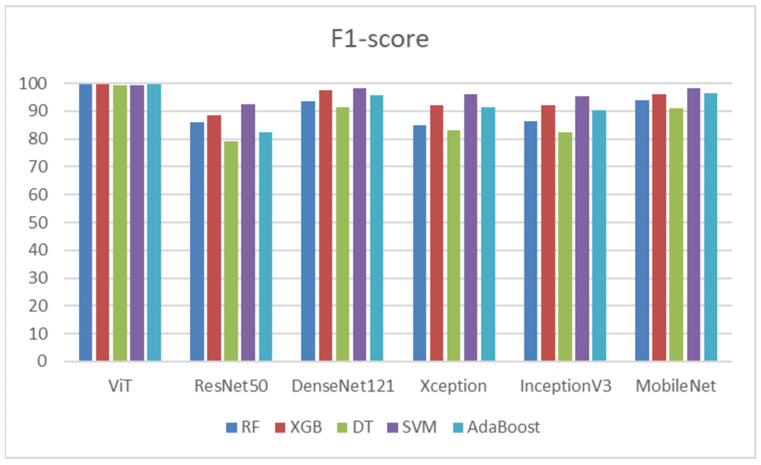
The F1-score of the five ML models.4.4. Discussion of the Proposed CAD Model.

From Table 16, the ViT model demonstrated exceptional F1-scores across all classifiers, making it the top performer. DenseNet121 and MobileNet also showed impressive results, particularly with the SVM classifier, which achieved scores exceeding 98%. In contrast, ResNet50 had the lowest F1-scores, especially with DT and AdaBoost. Xception and InceptionV3 exhibited moderate performance, with SVM generally producing higher F1-scores in both cases. Overall, the ViT model proved the most effective for achieving high F1-scores across all classifiers, followed closely by DenseNet121 and MobileNet, while ResNet50 lagged behind the others. Figure 16 shows the F1-scores of the five ML models.

TB is a contagious bacterial infection that primarily affects the lungs but can also impact other areas of the body, such as the brain, bones, and kidneys. The fight against TB is complicated by issues like drug resistance, simultaneous infections, and limited resources in heavily affected regions, which slow down efforts to eliminate the disease. Accurately and promptly detecting TB is crucial for effective management, as the disease often goes unnoticed and untreated, especially in remote and underprivileged areas. To address these challenges, we proposed a new hybrid CAD model. This innovative hybrid CAD approach combined ViT with PCA and ML techniques for TB classification, introducing a novel method in this field. In the hybrid CAD system, ViT was used as a base model for deep feature extraction, PCA was employed to reduce feature dimensions, and various ML methods (RF, XGB, DT, SVM, and AdaBoost) were used to classify TB cases. This system allowed for the prompt identification of TB, enabling timely medical intervention and improving patient outcomes.

Additionally, it streamlined the diagnostic process, reducing time and costs for patients and alleviating the workload on healthcare professionals. The CAD system can be integrated as a decision-support tool within existing radiology workflows. It serves as a secondary opinion system, flagging cases for further examination by radiologists rather than making standalone diagnoses.

We conducted two experiments. In the first experiment, we used transfer learning to pre-train ViT, ResNet50, DenseNet121, Xception, InceptionV3, and MobileNet. After pre-training, the models were fine-tuned using the training data from the TB Chest X-ray dataset. The results showed that ViT outperformed the other models across various evaluation metrics, achieving the highest average values for accuracy 99.4%, specificity 98.76%, NPV 99.05%, precision 99.05%, and F1-score 98.905%. Additionally, ViT demonstrated the lowest average FNR at 1.235%.

We also utilized transfer learning in the second experiment to pre-train the six DL models. The first step was supervised pre-training with the ImageNet dataset to extract deep features from the CXR images. Next, we fine-tuned five ML models (RF, XGB, DT, SVM, and AdaBoost) with each of the six DL models using the training data from the TB Chest X-ray dataset. During this second experiment, we employed each one of the six DL models as a base model to extract deep features, used PCA to reduce the dimensions of these features, and applied the ML methods (RF, XGB, DT, SVM, and AdaBoost) to classify the TB cases. The results showed that integrating ViT, PCA, and classifiers, such as RF, XGB, or AdaBoost, showcased exceptional performance across various evaluation metrics. It achieved the highest average values for accuracy 99.84%, specificity 99.52%, NPV 99.90%, precision 99.90%, recall 99.52%, and F1-score 99.71%. Additionally, it demonstrated the lowest average FNR at 0.48%.

The achieved FNR of 0.48% demonstrates the system’s strong capability to accurately identify positive cases, essential for reducing missed diagnoses, especially in critical conditions like TB. This low FNR enhances the system’s reliability as a diagnostic tool, ensuring that most TP cases receive prompt medical attention. However, despite the low rate of FN, there is a risk of delayed diagnosis and treatment, which could negatively impact patient outcomes and increase the likelihood of transmission of communicable diseases such as TB.

It is important to note that the CAD system is designed to assist, not replace, human expertise. All negative cases identified by the system should still be subject to clinical evaluation, particularly for high-risk patients or cases that are not clear-cut. Implementing thresholds based on patient history, symptoms, and other risk factors could prompt further testing or specialist consultations for borderline cases. Additionally, continuously learning from FN cases by retraining the model with new data can help minimize these errors over time.

The comparative analysis of the studies detailed in Table 17 reveals that Visuña1et al. [20] and Sharma et al. [22] achieved the highest accuracy rates of 99% and 99.29%, respectively. Visuña1et al. [20] employed a stacking and voting approach using VGG16, VGG19, and ResNet101V2 models, which performed nearly as well, reaching 99% accuracy. Sharma et al. [22] attained 99.29% accuracy using a segmentation-based approach (UNet) with the Xception model for classification.

However, the proposed hybrid model that integrated ViT with PCA and classifiers such as RF, XGB, or AdaBoost stands out with the highest classification accuracy of 99.84%. This method benefited from combining a DL model (ViT) with traditional ML techniques, capturing rich feature representations while optimizing the classification process.

ViT models have demonstrated exceptional performance in TB classification tasks, with the standalone ViT model achieving 99.4% accuracy and the hybrid model slightly outperforming it at 99.84%. This highlights the advantage of transformer-based architectures in capturing complex visual patterns. Finally, combining ViT with traditional RF, XGB, or AdaBoost techniques yields the best TB chest X-ray classification performance. These hybrid approaches leverage the strengths of different models to enhance accuracy and robustness across diverse datasets.

## 5. Conclusions

In this research, we introduced a hybrid CAD system that combines ViT with PCA and ML techniques for TB classification. In this hybrid CAD system, the ViT served as the base model for deep feature extraction, PCA was utilized to reduce the dimensions of the features, and various ML methods were applied to classify TB. This system enables rapid identification of TB, facilitating timely medical intervention and improving patient outcomes. Furthermore, it streamlines the diagnostic process, reducing both time and costs for patients while alleviating the workload on healthcare professionals. The CAD system can be incorporated as a decision-support tool within current radiology workflows. It acts as a secondary opinion system, highlighting cases for additional review by radiologists instead of providing independent diagnoses. The hybrid CAD system was developed and tested using a TB Chest X-ray dataset containing 4200 samples across two classes. ViT consistently demonstrated high accuracy across all classifiers, achieving results between 99.68% and 99.84%. This highlights ViT’s effectiveness with most classifiers, particularly with RF, XGB, and AdaBoost, which reached the highest accuracy of 99.84%. The CAD system was developed using the TB Chest X-ray dataset for training and evaluation. Before analysis, the dataset underwent preprocessing techniques such as resizing, scaling, and noise removal to enhance diagnostic accuracy. The CAD model exhibited impressive performance metrics, including an average precision of 99.90%, recall of 99.52%, F1-score of 99.71%, accuracy of 99.84%, FNR of 0.48%, specificity of 99.52%, and NPV of 99.90%. These assessments underscore the superior accuracy achieved by fine-tuning our model compared to existing classification tools. The current computational speed of the CAD presents a limitation. In our future projects, we will concentrate on implementing the Reinforced Transformer Network (RTN). This advanced method integrates the benefits of reinforcement learning with transformer models to evaluate image quality more efficiently. This strategy is especially beneficial for enhancing the diagnostic quality of Health Indicators), which are essential for identifying TB.

## Figures and Tables

**Table 1:** The sources of the TB Chest X-ray dataset.

Set	TB Images	Normal Images
NLM dataset	336	324
Belarus dataset	169	137
RSNA dataset	195	3039
**Total**	**700**	**3500**

**Table 2:** The TB Chest X-ray dataset.

Set	TB Images	Normal Images
Training	563	2797
Test	137	703
**Total**	**700**	**3500**

**Table 3:** The hyper-parameters values for the proposed fine-tuned CAD model.

Parameter	Value
img_size	224 × 224
Number of epoch	100
Batch size	256
num_heads	4
transformer_layers	8
mlp_head_units	[2048, 1024]
LayerNormalization	epsilon = 1 × 10^−6^
Activation	GELU
Optimizer	AdamW
Decay	0.0001
Dropout	0.5

**Table 4:** The evaluated measures for the ViT model.

Class	Accuracy (%)	Specificity (%)	FNR (%)	NPV (%)	Precision (%)	Recall (%)	F1-Score (%)
Normal	99.4	97.81	0.28	98.53	99.57	99.72	99.64
TB	99.4	99.72	2.19	99.57	98.53	97.81	98.17
**Average**	**99.4**	**98.765**	**1.235**	**99.05**	**99.05**	**98.765**	**98.905**

**Table 5:** The evaluated measures for the DenseNet121 model.

Class	Accuracy (%)	Specificity (%)	FNR (%)	NPV (%)	Precision (%)	Recall (%)	F1-Score (%)
Normal	99.29	97.08	0.28	98.52	99.43	99.72	99.57
TB	99.29	99.72	2.92	99.43	98.52	97.08	97.79
**Average**	**99.29**	**98.4**	**1.6**	**98.975**	**98.975**	**98.4**	**98.68**

**Table 6:** The evaluated measures for the MobileNet model.

Class	Accuracy (%)	Specificity (%)	FNR (%)	NPV (%)	Precision (%)	Recall (%)	F1-Score (%)
Normal	97.98	90.51	0.57	96.88	98.17	99.43	98.8
Tuberculosis	97.98	99.43	9.49	98.17	96.88	90.51	93.58
**Average**	**97.98**	**94.97**	**5.03**	**97.525**	**97.525**	**94.97**	**96.19**

**Table 7:** The evaluated measures for the ResNet101V2 model.

Class	Accuracy (%)	Specificity (%)	FNR (%)	NPV (%)	Precision (%)	Recall (%)	F1-Score (%)
Normal	97.38	96.35	2.42	88.59	99.28	97.58	98.42
Tuberculosis	97.38	97.58	3.65	99.28	88.59	96.35	92.31
**Average**	**97.38**	**96.965**	**3.035**	**93.935**	**93.935**	**96.965**	**95.365**

**Table 8:** The evaluated measures for the Xception model.

Class	Accuracy (%)	Specificity (%)	FNR (%)	NPV (%)	Precision (%)	Recall (%)	F1-Score (%)
Normal	98.33	91.24	0.28	98.43	98.32	99.72	99.01
Tuberculosis	98.33	99.72	8.76	98.32	98.43	91.24	94.7
**Average**	**98.33**	**95.48**	**4.52**	**98.375**	**98.375**	**95.48**	**96.855**

**Table 9:** The evaluated measures for the InceptionV3 model.

Class	Accuracy (%)	Specificity (%)	FNR (%)	NPV (%)	Precision (%)	Recall (%)	F1-Score (%)
Normal	98.81	96.35	0.71	96.35	99.29	99.29	99.29
Tuberculosis	98.81	99.29	3.65	99.29	96.35	96.35	96.35
**Average**	**98.81**	**97.82**	**2.18**	**97.82**	**97.82**	**97.82**	**97.82**

**Table 10:** The classification accuracy of the five ML models based on different deep feature extraction.

Deep Features/ML Classifier	RF	XGB	DT	SVM	AdaBoost
ViT	99.84	99.84	99.68	99.68	99.84
ResNet50	93.33	94.29	88.25	96.19	91.11
DenseNet121	96.67	98.57	95.24	99.05	97.62
Xception	93.02	96.03	90.63	97.94	95.56
InceptionV3	93.49	95.87	90.32	97.46	94.76
MobileNet	96.83	97.94	95.08	99.05	98.10

**Table 11:** The comparison of specificity of the five ML models based on different deep feature extraction.

Deep Features/ML Classifier	RF (%)	XGB (%)	DT (%)	SVM (%)	AdaBoost (%)
ViT	99.52	99.52	99.43	99.05	99.52
ResNet50	81.14	84.38	79.62	88.95	79.81
DenseNet121	90.38	96.48	90.67	97.52	95.14
Xception	79.05	88.48	83.33	93.81	88.57
InceptionV3	81.24	89.90	82.00	93.52	88.86
MobileNet	90.86	94.57	89.81	97.14	95.81

**Table 12:** The FNR of the five ML models.

Deep Features/ML Classifier	RF (%)	XGB (%)	DT (%)	SVM (%)	AdaBoost (%)
ViT	0.48	0.48	0.57	0.95	0.48
ResNet50	18.86	15.62	20.38	11.05	20.19
DenseNet121	9.62	3.52	9.33	2.48	4.86
Xception	20.95	11.52	16.67	6.19	11.43
InceptionV3	18.76	10.10	18.00	6.48	11.14
MobileNet	9.14	5.43	10.19	2.86	4.19

**Table 13:** The NPV of the five ML models.

Deep Features/ML Classifier	RF (%)	XGB (%)	DT (%)	SVM (%)	AdaBoost (%)
ViT	94.5	94.25	93.5	96.8	94.55
ResNet50	95.6	94.8	64.2	98.8	86.4
DenseNet121	98.8	98	87.1	99	94.1
Xception	100	98.8	71.7	100	94.2
InceptionV3	97.1	93.4	71.6	96.8	87.5
MobileNet	98.8	97.9	87.8	100	96

**Table 14:** The precision of the five ML classifiers based on six different deep feature extraction models.

Deep Features/ML Classifier	RF (%)	XGB (%)	DT (%)	SVM (%)	AdaBoost (%)
ViT	99.90	99.90	99.43	99.81	99.90
ResNet50	94.35	94.51	78.75	97.30	86.19
DenseNet121	97.58	98.34	91.96	99.03	96.21
Xception	96.13	97.20	83.08	98.79	95.01
InceptionV3	95.06	94.85	82.75	97.21	91.78
MobileNet	97.68	97.93	92.09	97.68	97.26

**Table 15:** The recall of the five ML classifiers.

Deep Features/ML Classifier	RF (%)	XGB (%)	DT (%)	SVM (%)	AdaBoost (%)
ViT	99.52	99.52	99.43	99.05	99.52
ResNet50	81.14	84.38	79.62	88.95	79.81
DenseNet121	90.38	96.48	96.48	97.52	95.14
Xception	79.05	88.48	83.33	93.81	88.57
InceptionV3	81.24	89.90	82.00	93.52	88.86
MobileNet	90.86	94.57	89.81	97.14	95.81

**Table 16:** The F1-score of the five ML classifiers.

Deep Features/ML Classifier	RF (%)	XGB (%)	DT (%)	SVM (%)	AdaBoost (%)
ViT	99.71	99.71	99.43	99.42	99.71
ResNet50	86.00	88.44	79.17	92.50	82.49
DenseNet121	93.52	97.38	91.30	98.26	95.66
Xception	84.74	92.15	83.21	96.09	91.40
InceptionV3	86.26	92.15	82.37	95.25	90.23
MobileNet	93.86	96.15	90.90	98.25	96.52

**Table 17:** The comparison of our CAD with the state-of-the-art.

Reference	Methodology	Performance	Dataset
Acharya et al. [4]	Normalization-free network and RandAugment,	96.91% on TBX11K	Set 1(TB chest X-ray, Montgomery, Shenzhen, and DatasetA + DatasetB) Set2 (Belarus, NIAID, and RSNA)
		95.91% on Kaggle dataset	
Huy and Lin [10]	Convolutional Block Attention Module (CBAM) and the Wide Dense Net (WDnet)	98.80% on TBX11K	Total DS (TB chest X-ray, Montgomery, and Shenzhen)
		96.80% Total DS	
Le et al. [19]	VGG16, EfficientNet-B7, MobileNetV3, DenseNet121, and RegNetY040	MobileNetV3 at 98.35%	TB chest X-ray
		77.81%	Montgomery
		67.19%	Shenzhen
Visuña1et al. [20]	CNN-ensembles: Stacking and Voting (VGG16, VGG19, InceptionV3, ResNet101V2, DenseNet121 and CheXnet) and Grad-CAM.	99% for stacking and 98% for voting	TB chest X-ray
Goswami et al. [21]	CNN	94%	TB chest X-ray
Sharma et al. [22]	UNet segmentation model, Xception model, and The Grad-CAM	96.35% for segmentation and 99.29% for classification	Montgomery and Shenzhen for segmentation and TB chest X-ray for classification
Maheswari et al. [23]	shallow- CNN architecture and Bayesian optimization technique	95%	TB chest X-ray
**Proposed CAD model**	**ViT**	**99.4%**	**TB chest X-ray**
**Proposed Hybrid CAD model**	**ViT, PCA and (RF, XGB, or AdaBoost)**	**99.84%**	**TB chest X-ray**

## Data Availability

All information utilized in this research is available upon request. The data include the dataset Tuberculosis (TB) chest X-ray dataset sourced from Kaggle, https://www.kaggle.com/tawsifurrahman/tuberculosis-tb-chest-xray-dataset [last accessed on 2 July 2024].
